# CRISPRi-mediated characterization of novel anti-tuberculosis targets: Mycobacterial peptidoglycan modifications promote beta-lactam resistance and intracellular survival

**DOI:** 10.3389/fcimb.2023.1089911

**Published:** 2023-03-15

**Authors:** Cátia Silveiro, Mariana Marques, Francisco Olivença, David Pires, Diana Mortinho, Alexandra Nunes, Madalena Pimentel, Elsa Anes, Maria João Catalão

**Affiliations:** ^1^ Research Institute for Medicines (iMed.ULisboa), Faculty of Pharmacy, Universidade de Lisboa, Lisboa, Portugal; ^2^ Universidade Católica Portuguesa, Católica Medical School, Centre for Interdisciplinary Research in Health, Lisbon, Portugal; ^3^ Genomics and Bioinformatics Unit, Department of Infectious Diseases, National Institute of Health, Lisbon, Portugal; ^4^ Faculty of Veterinary Medicine, Universidade Lusófona, Lisbon, Portugal

**Keywords:** tuberculosis, antibiotic resistance, peptidoglycan modifications, CRISPR interference, beta-lactams, intracellular survival, host-pathogen interactions, anti-TB targets

## Abstract

The lack of effective therapeutics against emerging multi-drug resistant strains of *Mycobacterium tuberculosis* (*Mtb*) prompts the identification of novel anti-tuberculosis targets. The essential nature of the peptidoglycan (PG) layer of the mycobacterial cell wall, which features several distinctive modifications, such as the *N*-glycolylation of muramic acid and the amidation of D-*iso*-glutamate, makes it a target of particular interest. To understand their role in susceptibility to beta-lactams and in the modulation of host-pathogen interactions, the genes encoding the enzymes responsible for these PG modifications (*namH* and *murT*/*gatD*, respectively) were silenced in the model organism *Mycobacterium smegmatis* using CRISPR interference (CRISPRi). Although beta-lactams are not included in TB-therapy, their combination with beta-lactamase inhibitors is a prospective strategy to treat MDR-TB. To uncover synergistic effects between the action of beta-lactams and the depletion of these PG modifications, knockdown mutants were also constructed in strains lacking the major beta-lactamase of *M. smegmatis* BlaS, PM965 (*M. smegmatis* Δ*blaS1*) and PM979 (*M. smegmatis* Δ*blaS1* Δ*namH*). The phenotyping assays affirmed the essentiality of the amidation of D-*iso*-glutamate to the survival of mycobacteria, as opposed to the *N*-glycolylation of muramic acid. The qRT-PCR assays confirmed the successful repression of the target genes, along with few polar effects and differential knockdown level depending on PAM strength and target site. Both PG modifications were found to contribute to beta-lactam resistance. While the amidation of D-*iso*-glutamate impacted cefotaxime and isoniazid resistance, the *N*-glycolylation of muramic acid substantially promoted resistance to the tested beta-lactams. Their simultaneous depletion provoked synergistic reductions in beta-lactam MICs. Moreover, the depletion of these PG modifications promoted a significantly faster bacilli killing by J774 macrophages. Whole-genome sequencing revealed that these PG modifications are highly conserved in a set of 172 clinical strains of *Mtb*, demonstrating their potential as therapeutic targets against TB. Our results support the development of new therapeutic agents targeting these distinctive mycobacterial PG modifications.

## 1 Introduction

*Mycobacterium tuberculosis* (*Mtb*), the etiologic agent of tuberculosis (TB), latently infects one-quarter of the world’s population and causes about 1.5 million deaths annually (WHO, 2021). The emergence and spread of multi-drug (MDR) and extensively-drug resistant (XDR) strains of *Mtb* has accentuated the demand for novel vaccine candidates and for improved therapeutic regimens (Singh and Chibale, 2021; WHO, 2021). Therefore, the development of innovative anti-TB agents, which depends on the identification of novel drug targets, is imperative (Singh and Chibale, 2021).

The notable success of *Mtb* as a pathogen relies on its unusual cell wall (CW), comprising a heavily cross-linked and modified network of peptidoglycan (PG), a highly branched arabinogalactan (AG) polysaccharide and typical long-chain mycolic acids (MA) (Jankute et al., 2015; Catalão et al., 2019; Maitra et al., 2019). The mycobacterial PG features unique modifications, of which the *N*-glycolylation of muramic acid and the amidation of D-*iso*-glutamate are of particular significance (Catalão et al., 2019; Maitra et al., 2019).

Unlike most bacteria, the PG from *Mycobacterium* and related genera consists of alternating residues of *N*-acetylglucosamine and a mixture of muramic acid residues, encompassing *N*-glycolyl and *N*-acetyl modifications (Mahapatra et al., 2005; Raymond et al., 2005). The *N*-glycolylation of muramic acid, catalyzed by the *N*-acetyl muramic acid hydroxylase (NamH), occurs when the *N*-acetyl function is converted to the *N*-glycolyl function, generating *N*-glycolyl muramic acid (Mur*N*Glyc) (Raymond et al., 2005). Previous studies have probed the role of this PG modification in antibiotic susceptibility (Raymond et al., 2005; Coulombe et al., 2009) and in host-pathogen interactions (Coulombe et al., 2009; Hansen et al., 2014; Wang et al., 2016), inferring that the *N*-glycolylation of PG promotes resistance to beta-lactams and lysozyme (Raymond et al., 2005) and potentiates the immunogenicity of the mycobacterial CW (Coulombe et al., 2009; Hansen et al., 2014).

Another distinctive mycobacterial PG modification is the amidation of the free carboxyl group of the D-*iso*-glutamate (D-*iso*-Glu) residue at the second position of both lipid II and its precursor lipid I (Catalão et al., 2019; Maitra et al., 2019). This PG modification, catalyzed by the MurT/GatD amidotransferase complex, has been characterized in *Staphylococcus aureus* (Figueiredo et al., 2012; Münch et al., 2012) and in *Streptococcus pneumoniae* (Morlot et al., 2018). This process begins with the hydrolysis of L-glutamine to generate glutamate and ammonia, catalyzed by GatD (Figueiredo et al., 2012; Münch et al., 2012; Leisico et al., 2018; Morlot et al., 2018). The ammonia is then used to catalyze the amidation of D-*iso*-Glu into D-*iso*-glutamine by MurT, a Mur ligase-like protein, in an ATP and Mg^2+^-dependent manner. The inhibition of D-*iso*-Glu amidation in *S. aureus* seems to delay cell division and to increase susceptibility to beta-lactams and lysozyme (Figueiredo et al., 2012; Münch et al., 2012; Figueiredo et al., 2014). Moreover, D-*iso*-Glu amidated lipid II has been postulated as the preferred substrate for transpeptidation reactions in *S. aureus* (Figueiredo et al., 2012; Münch et al., 2012), *S. pneumoniae* (Zapun et al., 2013) and *Mtb* (Pidgeon et al., 2019). Recently, the enzymatic activity of the MurT/GatD complex has been characterized in *Mtb*, revealing the co-transcription of the *murT*/*gatD* operon and the ATPase activity of MurT (Maitra et al., 2021).

Despite being a successful and relatively safe antibiotic class, beta-lactams have not yet been included in TB-therapeutic regimens because of the inherent resistance of *Mtb* to this class, fundamentally attributed to a chromosomally encoded beta-lactamase, the impermeable nature of the mycobacterial CW and the predominance of non-classical PG cross-links, only efficiently inhibited by carbapenems (Cordillot et al., 2013; Wivagg et al., 2014; Story-Roller and Lamichhane, 2018). Whereas *Mtb* encodes a very efficient beta-lactamase BlaC, *M. smegmatis* produces both a highly active beta-lactamase BlaS and a lowly active cephalosporinase BlaE (Flores et al., 2005). The predominance of non-classical 3→3 cross-links in mycobacterial PG is attributed to the action of L, D - transpeptidases (Ldts) while penicillin binding proteins (PBPs) catalyze the usual 4→3 cross-links (Lavollay et al., 2008; Kumar et al., 2012; Cordillot et al., 2013; Baranowski et al., 2018). Nevertheless, several studies have encouraged the repurposing of carbapenems in combination with beta-lactamase inhibitors (Hugonnet and Blanchard, 2007) to treat MDR-TB infections (Hugonnet et al., 2009; England et al., 2012; De Lorenzo et al., 2013; Cohen et al., 2016; Zhang et al., 2016). Additionally, the World Health Organization (WHO) currently endorses two carbapenems in group C of the drugs used to treat MDR-TB patients on longer therapeutic regimens (WHO, 2020). Recently, our group identified several drivers of resistance/susceptibility to beta-lactams in *Mtb* (Olivença et al., 2022a; Olivença et al., 2022b), alluding to the potential introduction of beta-lactams in TB therapeutic schemes.

So far, the lack of efficient gene inactivation tools for mycobacteria has hindered the identification of novel vaccine candidates and drug targets. The discovery of CRISPR-Cas (Clustered Regularly Interspaced Short Palindromic Repeats-CRISPR associated proteins) systems has facilitated precise genome editing (Doudna and Charpentier, 2014). CRISPR has recently been repurposed for bacterial transcription regulation through the inactivation of Cas9 by point mutations within its nuclease domains, generating an endonuclease-deficient Cas9 (dCas9) with preserved DNA-binding activity (Bikard et al., 2013; Larson et al., 2013; Qi et al., 2013). In *Escherichia coli*, CRISPR interference (CRISPRi) employs a dCas9-sgRNA complex to precisely repress any target gene by steric hindrance (Bikard et al., 2013; Qi et al., 2013). The system’s target specificity is determined by the complementarity between the sgRNA and the target sequence and by the presence of a short motif downstream the target sequence, the protospacer adjacent motif (PAM) (Mojica et al., 2009). CRISPRi can prevent transcription initiation whether the promoter region is targeted or block mRNA elongation by RNA polymerase whether the coding sequence is targeted (Bikard et al., 2013; Larson et al., 2013; Qi et al., 2013). To achieve a high knockdown efficiency, the non-template strand of a gene or both strands of its promoter can be targeted (Qi et al., 2013). Several reports have established the use of anhydrotetracycline (ATc)-inducible (Ehrt et al., 2005) CRISPRi systems in mycobacteria, however with associated toxicity and producing a low level of knockdown (Choudhary et al., 2015; Singh et al., 2016). More recently, an ATc-inducible CRISPRi system based on the use of Cas9 from *Streptococcus thermophilus* (dCas9_Sth1_), employing several PAM variants, was developed (Rock et al., 2017).

In this work, we implement the dCas9_Sth1_-based CRISPRi system in *M. smegmatis* to assert the role of the characteristic modifications of mycobacterial PG, catalyzed by the NamH hydroxylase and the MurT/GatD complex, in susceptibility to beta-lactams and in host-pathogen interactions.

## 2 Materials and methods

### 2.1 Bacterial strains, plasmids, cell lines, culture conditions and antibiotics

The bacterial strains, plasmids and cell lines used in this study are listed in **Table 1**. To amplify the CRISPRi backbone PLJR962 (Addgene #115162) and for cloning, *E. coli* was cultured in Luria-Bertani medium (Merck), supplemented with 25 μg/mL of kanamycin (NZYTech) when appropriate. *M. smegmatis* strains were cultured in Middlebrook 7H9 broth (BD™ Biosciences) supplemented with 0.2% glycerol (ThermoScientific), 0.5% glucose (Sigma-Aldrich) and 0.05% tyloxapol (Sigma-Aldrich) or 7H10 agar (BD™ Biosciences) supplemented with 0.5% glycerol. When necessary, the medium was supplemented with 25 μg/mL of kanamycin. Bacteria were grown at 37°C with (broth) or without (agar) shaking at 180 rpm. To induce sgRNA and dCas9_Sth1_ expression, 100 ng*/*mL of ATc were added to mycobacterial cultures every 24 h. The J774.A1 cells were cultured in DMEM media (Gibco) supplemented with 10% fetal bovine serum, 1% sodium pyruvate, 1% L-Glutamine, and 1% HEPES buffer. The growth of J774.A1 cells occurred at 37°C, with 5% CO_2_. Stocks of amoxicillin (AMX), cefotaxime (CTX), ethambutol (EMB), meropenem (MEM) and isoniazid (INH) (Sigma-Aldrich) were prepared with purified MilliQ water. ATc (Sigma-Aldrich) was prepared at a stock concentration of 10 mg/mL in dimethyl sulfoxide (DMSO) cell culture grade (AppliChem). Potassium clavulanate (CLA) (Sigma-Aldrich) was prepared in phosphate buffer pH 6.0, 0.1 M.

**Table 1:** Bacterial strains, plasmids and cell lines used in this study.

Bacterial strain, plasmid or cell line	Description	Reference or source
Bacteria		
*Escherichia coli*		
JM109	*recA1 endA1 gyr96 thi hsdR17 supE44 relA1* Δ(*lac-proAB*) [F´ *traD36 proAB lac* ^q^ *Z*ΔM15]	Stratagene
*Mycobacterium smegmatis*		
mc^2^ 155 (WT)	High-transformation-efficiency mutant of *M. smegmatis* ATCC 607	Snapper et al., 1988
PM965	*M. smegmatis* mc^2^ 155 *ept-1 rpsL4 ΔblaS*1	Raymond et al., 2005
PM979	*M. smegmatis* mc^2^ 155 *ept-1 rpsL4 ΔblaS1 ΔnamH*	Raymond et al., 2005
Plasmids		
PLJR962 (8881 bp)	Sth1 *dcas9* Tet^R^ and Kan^R^ L5 Int *attP* for *M. smegmatis*	Rock et al., 2017
Cell lines		
J774.A1	Murine-derived monocyte/macrophage cell line	ATCC, TIB-67

### 2.2 Construction of knockdown mutants using CRISPRi

The cloning procedures were executed as previously optimized (Rock et al., 2017; Marques, 2020; Silveiro, 2020; Wong and Rock, 2021). To achieve efficient repression, either the non-template strand or the promoter region of the target genes *murT* (*MSMEG_6276*), *gatD* (*MSMEG_6277*) and *namH* (*MSMEG_6410*) were targeted. Various PAM sequences were identified on the template strand of the target genes, chosen for differential repression regarding PAM strength and target site, and accordingly guided sgRNA design (**Table S1**). The CRISPRi backbone PLJR962 was amplified in *E. coli* JM109 and 2 μg of plasmid were digested with BsmB*I* (ThermoScientific) in the presence of 1 mM DTT (ThermoScientific) for 4 h at 37°C in a PCR block (Applied Biosystems Veriti™). After, the digestion reactions were purified using the QIAquick PCR Purification Kit (Qiagen). To clone the sgRNA targeting sequences into the sgRNA scaffold, two complementary primers were designed (**Table S2**), synthesized (Eurofins Genomics), annealed, and ligated into PLJR962 using T4 DNA ligase (400 U/µL; NEB) at 22°C for 30 min. To obtain the sgRNA-containing CRISPRi plasmids, *E. coli* chemically competent cells were transformed with the ligation mixture using a double heat-shock approach, followed by plasmid extraction from the obtained recombinants with the NZYMiniprep kit (NZYTech). The obtainment of the desired sgRNA-containing CRISPRi plasmids was confirmed by Sanger Sequencing. To express the recombinant clones as knockdown mutants in *M. smegmatis*, competent cells were transformed with 500 ng of recombinant plasmid by electroporation (using 2 mm cuvettes and a Bio-Rad Gene Pulser™ Electroporation System, with pulse settings of 2.5 kV, 1000 Ω resistance and 25 μF capacitance) in the presence of glycerol 10% (Goude and Parish, 2009; Marques, 2020; Silveiro, 2020).

### 2.3 Phenotyping

To evaluate differences in cell viability after CRISPRi induction, spotting assays were performed as previously described (Marques, 2020; Silveiro, 2020). Briefly, cultures of *M. smegmatis* were grown to log phase, normalized to a theoretical OD_600_ of 0.001 and, serially diluted in a 96-well plate. A minimal quantity (5 µL) of each bacterial suspension was plated on solid media with and without ATc and incubated at 37°C for 2 days. At least three biological replicates were performed.

### 2.4 RNA extraction

Bacterial cultures were grown to log phase, normalized to a theoretical OD_600_ of 0.001 and, incubated at 37°C with shaking until an OD_600_ of 0.1 was reached (Marques, 2020; Silveiro, 2020; Wong and Rock, 2021). After an incubation period of 6 h with or without ATc, the cultures were harvested by centrifugation at 3000 g for 5 min, ressuspended in 500 µL of RNAprotect Bacteria Reagent (Qiagen) and, stored at -20°C. When convenient, the cells were thawed and pelleted at 5000 g for 5 min at 4°C. Lysis was promoted by the addition of 350 µL of buffer NR (NZYTech) and 3.5 µL of beta-mercaptoethanol (Merck), followed by intense vortexing. The cells were then disrupted in a BeadBug™ (Benchmark Scientific) by 4 cycles at maximum speed for 1 min, interspersed by resting periods of 1 min on ice. After a spin-down, the lysate was applied into the NZYSpin Homogenization column, and the subsequent steps were performed according to the instructions of the NZYTotal RNA Isolation Kit (NZYTech). The RNA was eluted in 40 µL of DEPC water (Invitrogen) at 60°C. Genomic DNA contamination was avoided by performing a rigorous treatment with TurboDNase (Invitrogen). The quantity and purity of the obtained RNA was determined using Nanodrop™ and the absence of significant DNA contamination was confirmed by performing a PCR on the housekeeping gene *sigA* using NZYTaq DNA polymerase (NZYTech) followed by 1.5% agarose gel electrophoresis.

### 2.5 Quantitative reverse-transcription PCR

Purified RNA (100 ng) was used to synthesize cDNA (Singh et al., 2016) by reverse transcription using the NZY First-Strand cDNA Synthesis Kit (NZYTech). Subsequently, the cDNA levels were quantified by qPCR using the NZYSupreme qPCR Green Master Mix (2x), ROX (NZYTech) and a QuantStudio™ 7 Flex Real-Time PCR System (Applied Biosystems). The qRT-PCR primers were designed using Primer 3^1^ to amplify a specific product between 100-250 bps (**Table S3**). The PCR reaction (40 cycles) proceeded as follows: 95°C for 10 min, 95°C for 15 s, 60°C for 30 s. The amplification efficiency of the primers was verified to be 80-110%. For each run, at least two technical replicates and a negative control were produced, and the mean C_T_ was calculated. Data were analyzed using the Comparative C_T_ method (ΔΔC_T_ method; the fold change is calculated as 2^-ΔΔCT^) with *M. smegmatis* WT as the calibrator sample and *sigA* as a reference gene (Choudhary et al., 2015; Rock et al., 2017; Wong and Rock, 2021). Two biological replicates were utilized for the quantification of the target sequences (Singh et al., 2016).

### 2.6 Minimum inhibitory concentration determination

The antibiotic susceptibility determination assays were performed for three beta-lactams (amoxicillin, cefotaxime and meropenem), with and without the beta-lactamase inhibitor clavulanate, and for two antimycobacterial agents (isoniazid and ethambutol), as described previously (Marques, 2020; Silveiro, 2020). The antibiotics were prepared at the desired initial concentration (512 μg/mL for beta-lactams and 128 μg/mL for EMB/INH) while clavulanate was used at a fixed concentration of 2.5 μg/mL. Next, the antibiotics were serially diluted 1:2 in supplemented 7H9 media. *M. smegmatis* cultures were grown to log phase, diluted to a theoretical OD_600_ of 0.002 and split into two equal volumes with and without 100 ng/mL of ATc. Subsequently, 100 µL of each bacterial suspension was added to the respective wells and, the plates were incubated at 37°C for 3 days. After incubation, the wells were observed for bacterial growth, usually indicated by turbidity or the presence of a bacterial pellet in the bottom of the wells. The dilution at which no bacterial growth was observed corresponds to the annotated MIC value. This assay was performed at least three times for each culture of interest and the median MIC values were calculated. Whenever mentioned, the “breakpoints” refer to the EUCAST non-species related PK/PD breakpoints^2^ (version 12) because there are not any currently defined beta-lactam breakpoints for mycobacteria.

### 2.7 Macrophage infections and intracellular survival assessment

To address the contribution of PG modifications to the intracellular survival of mycobacteria, J774.A1 macrophages were infected with control and mutant strains, as described previously (Anes et al., 2006; Jordão et al., 2008). After several passages and 48 h before infection, the macrophages were seeded in 96-well plates at a density of 12.5 x 10^3^ cells/well. That day, bacterial cultures were diluted to the same OD_600_ and, incubated at 37°C with shaking until an OD_600_ of 0.1 was reached. At that point (approximately 24 h before infection), the cultures were incubated with or without 100 ng/mL of ATc. On the day of infection, the cultures were centrifuged at 3000 g for 5 minutes and washed with 5 mL of PBS 1 X (Gibco) (Anes et al., 2006; Jordão et al., 2008). To obtain a single-cell suspension, the cultures were resuspended in DMEM media, subjected to 5 min of ultrasonic bath, and centrifuged at 500 g for 1 min (Pires et al., 2017). The cell media was discarded from the seeding plates and the macrophages were infected with a single-cell suspension of mycobacteria at a multiplicity of infection (MOI; ratio of bacteria/cells) of 5 and, incubated at 37°C with 5% CO_2_ for different timepoints (1 h; 4 h; 24 h) (Anes et al., 2006; Jordão et al., 2008; Marques, 2020; Silveiro, 2020). Following internalization, the cells were washed twice with PBS 1 X and resuspended in DMEM supplemented with 10 μg/mL gentamicin to kill extracellular bacteria and 100 ng/mL of ATc, when appropriate.

At timepoints 1 h, 4 h and 24 h, the media was discarded, and the macrophages were lysed after 15 min incubation with a 0.05% IGEPAL solution (Pires et al., 2017). Serial dilutions of the resulting lysates were prepared in sterile water and plated onto 7H10 plates, which were incubated for 2 days at 37°C with 5% CO_2_ (Anes et al., 2006; Jordão et al., 2008). The colony forming units (CFUs) were then counted using an inverted microscope (Nikon TMS Inverted Phase Contrast Microscope). Three biological replicates, each counting three technical replicates, were performed.

### 2.8 Single nucleotide polymorphism analysis in clinical strains of *Mtb*

To assess whether the genes encoding the enzymes responsible for the characteristic modifications of mycobacterial PG were conserved in *Mtb*, data was retrieved from a study recently published by our group that set to uncover beta-lactam susceptibility patterns in a collection of 172 clinical strains of *Mtb* (Olivença et al., 2022b). Briefly, for each strain, quality improved reads were individually mapped against Mtb H37Rv reference genome (GenBank accession number AL123456.3), and the identification of single nucleotide polymorphisms (SNPs) was performed as previously described (Olivença et al., 2022b). The resulting MIC values were used in this study to uncover susceptibility differences to AMX or MEM, with or without clavulanate, that could be associated with frequent SNPs (with a mutation frequency ≥ 3) in the proposed genes of interest.

### 2.9 Statistical analysis

The statistical analysis was preformed using GraphPad (version 8.4.3.686) and data are presented as mean ± SEM, except if stated otherwise. Normality testing for qRT-PCR or CFUs data was preformed using Q-Q plots or the Shapiro-Wilk Test, respectively. Multiple comparisons were made using one-way ANOVA, followed by pairwise comparisons of selected groups using the Holm-Sidak test. All the prerequisites of the tests were verified.

## 3 Results

### 3.1 Construction of single PG modification knockdown mutants using CRISPR interference

**Figure 1:**
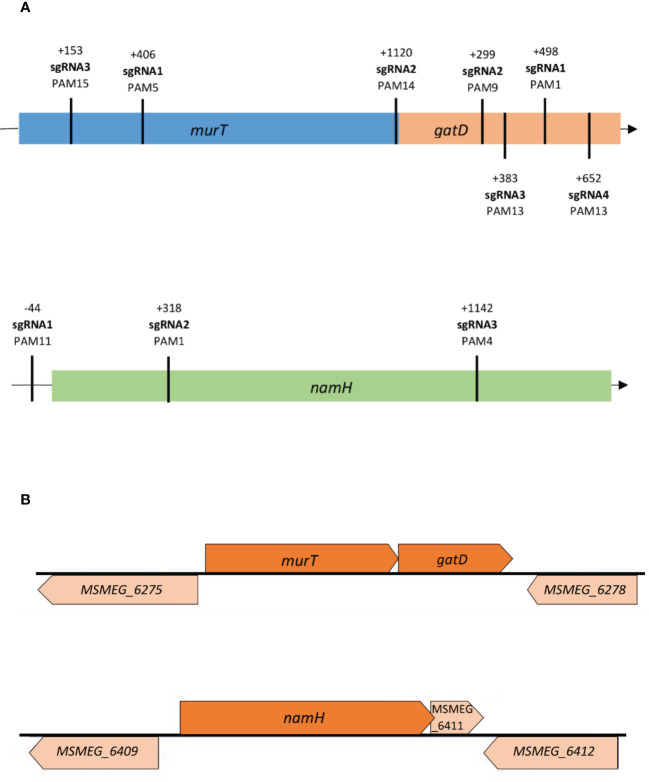
**(A)** Distribution of sgRNA targeting sequences along the *murT*/*gatD* operon and the *namH* gene in *M. smegmatis*. Different sites were targeted along the genes to assess the differential repression efficiency of CRISPRi; **(B)** The gene local context (illustration based on data from the Biocyc database) of the target genes *murT*, *gatD* and *namH* in *M. smegmatis* needs to be considered due to possible downstream/upstream polar effects. The *murT* and *gatD* genes are co-transcribed under regulation of a single promoter. The *MSMEG_6275* precedes the *murT* gene but is transcribed in the opposite direction while the *MSMEG_6278* gene occurs after the *gatD* gene but is also transcribed in the opposite direction. A single promoter guides the conjoint transcription of the *namH* and *MSMEG_6411* genes by RNA polymerase. The *MSMEG_6409* precedes the *namH* gene but is transcribed in the opposite direction while the *MSMEG_6412* gene occurs after the *MSMEG_6411* gene but is also transcribed in the opposite direction.

To construct the knockdown mutants, a previously developed ATc-inducible dCas9_Sth1_-based CRISPRi system, which enables the efficient knockdown of both essential and non-essential genes in mycobacteria, was used (Rock et al., 2017; Wong and Rock, 2021). To achieve efficient repression, either the non-template strand or the promoter region of the target genes *murT* (*MSMEG_6276*), *gatD* (*MSMEG_6277*) and *namH* (*MSMEG_6410*) were targeted. The efficiency of CRISPRi-mediated gene silencing is determined by several factors, namely PAM strength and the PAM position along the targeted gene (Rock et al., 2017; Marques, 2020). Therefore, various PAM sequences were identified on the template strand of the target genes and chosen for differential repression regarding PAM strength and target site (**Figure 1A**). PAM strength is directly proportional to the silencing efficiency, indicated as fold repression, of the possible PAM variants for dCas9_Sth1_-mediated gene silencing in mycobacteria (Rock et al., 2017; Wong and Rock, 2021). To avoid lethality effects when targeting allegedly essential genes, the essentiality classification of the respective orthologue genes in *Mtb* was considered. The gene local context was also taken into consideration due to the previously reported probability of downstream polar effects (Choudhary et al., 2015; Singh et al., 2016; Rock et al., 2017) and reverse polarity for operonic genes (Peters et al., 2016), as is the case of our target genes (**Figure 1B**).

#### 3.1.1 Phenotyping of the single mutants through spotting assays

**Figure 2:**
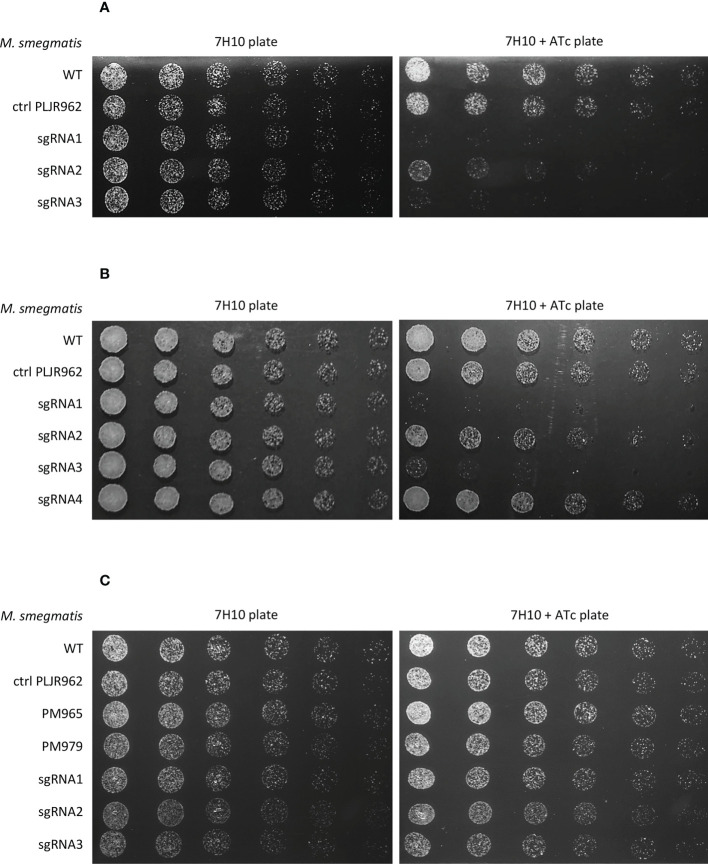
Spotting assays of all *M. smegmatis* knockdown mutants (n=3): **(A)** *murT* knockdown mutants; **(B)** *gatD* knockdown mutants; **(C)** *namH* knockdown mutants. Negative controls (*M. smegmatis* WT, PLJR962 and PM965) and a positive control (*M. smegmatis* PM979) were included in all experiments. The first spots were normalized to an OD_600_ of 0.001 and the next were performed by preparing and spotting 2-fold serial dilutions of each culture on agar plates.

To phenotypically characterize the knockdown mutants, spotting assays were performed. The addition of inducer did not influence the viability of the negative controls *M. smegmatis* WT and *M. smegmatis* PLJR962. The results from the spotting assays show that the *murT/gatD* operon is essential for the viability of *M. smegmatis* since growth defects were observed for most knockdown mutants in the presence of inducer (**Figure 2A, B**) (Marques, 2020). In the case of *murT* knockdown (*murT* ^-^), sgRNA1-*murT* and sgRNA3-*murT* were responsible for severe growth defects, while sgRNA2-*murT* only moderately affected bacterial viability. In the case of *gatD* knockdown (*gatD* ^-^), sgRNA1-*gatD* and sgRNA3-*gatD* provoked severe growth defects in the spotting assays whereas sgRNA2-*gatD* caused a slight viability defect and sgRNA4-*gatD* did not appear to affect viability.

MurT comprises two main domains: the first is homologous to the central domain of Mur ligases (Mur_Ligase_M), and the second is a C-terminal domain without known function (DUF1727) (Figueiredo et al., 2012; Münch et al., 2012; Gonçalves et al., 2019; Maitra et al., 2021). The sequence regions that encode the MurT central domain, involved in the binding of the essential cofactors ATP (K59, E108) and Mg^2+^ (Figueiredo et al., 2012; Maitra et al., 2021) are targeted by sgRNA1-*murT* and sgRNA3-*murT*. A sequence alignment of MurT from *S. aureus*, *S. pneumoniae*, *M. smegmatis and Mtb* shows that these sequences are conserved in both *MSMEG_6276* and its orthologue in *Mtb*, *Rv3712* (**Figure S1**) (Marques, 2020). The inhibition of transcription elongation by dCas9_Sth1_ can generate truncated transcripts, lacking these essential regions, thereby contributing to the low viability phenotype displayed by the respective knockdown mutants (Marques, 2020). The DUF1727 domain has four conserved amino acid regions (I, II, III and IV) that regulate the interaction between MurT and GatD (Gonçalves et al., 2019; Maitra et al., 2021). Although the target site of sgRNA2-*murT* codes for the DUF1727 domain, the targeted region corresponds to the final nucleotides of *murT*. Therefore, the RNA polymerase can transcribe almost the entire sequence of the *murT* gene, possibly leading to the production of a semi-functional protein and, thus, enabling the growth of the respective knockdown mutant (Marques, 2020). In contrast, GatD has a glutaminase domain similar to that of class I-type GATases, possessing conserved residues responsible for its catalytic glutamine amidotransferase activity in GatD (C94, H189) and in MurT (D349). GatD also possesses conserved catalytic residues involved in the sequestration of glutamine (R128) and in the channeling of ammonia to MurT (G59) (Figueiredo et al., 2012; Münch et al., 2012; Leisico et al., 2018; Maitra et al., 2021). A sequence alignment of GatD from *S. aureus*, *S. pneumoniae*, *M. smegmatis and Mtb* shows that these sequences are conserved in both *MSMEG_6277* and its orthologue in *Mtb*, *Rv3713* (**Figure S2**) (Marques, 2020). CRISPRi-mediated interference of *gatD* with sgRNAs 1 to 3, which target sites near these conserved residues, might result in depleted D-*iso*-Glu amidation (Marques, 2020). This effect is directly proportional to PAM strength and could explain the growth inhibition phenotypes observed with sgRNA1-*gatD* and sgRNA3-*gatD*.

Regarding the target site and PAM strength distribution of the employed sgRNAs, it was observed that the distance from the transcription start site (TSS) directly impacts the phenotype of these mutants, mainly when weak PAMs are employed (Marques, 2020). In the case of *murT*, both sgRNA2-*murT* and sgRNA3-*murT* have weak PAMs, however sgRNA3-*murT* (PAM15, +153 bp) produced considerably more severe growth defects than sgRNA2-*murT* (PAM14, +1120 bp), likely due to the target site. In the case of *gatD*, greater growth defects were observed with sgRNA3-*gatD* (PAM13, +383 bp) when compared to sgRNA4-*gatD* (PAM13, +652 bp), although they share the same PAM.

On the other hand, *namH* is not an essential gene for the viability of *M. smegmatis* since no growth defects were observed in the presence of inducer (**Figure 2C**) (Silveiro, 2020).

#### 3.1.2 Assessment of CRISPRi-mediated gene knockdown in the single mutants by qRT-PCR

**Figure 3:**
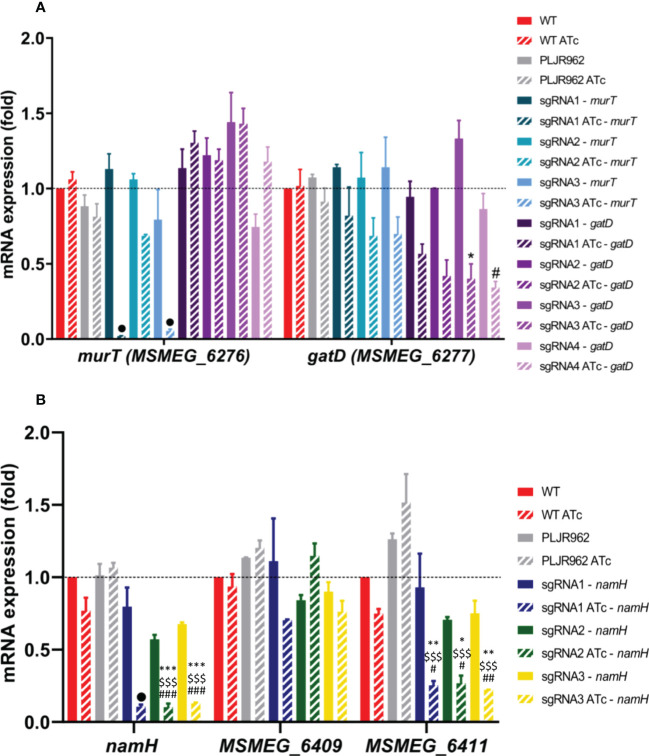
Results of qRT-PCR assays for the single mutants, constructed in the *M. smegmatis* WT strain (n=2): **(A)** *murT* (blue shade bars) and *gatD* (purple shade bars) knockdown mutants; **(B)** *namH* knockdown mutants. The graph shows the mean of the relative mRNA expression of genes of interest normalized to *sigA*, at 6 hours post-induction, with (stripped bars) and without (smooth bars) ATc. The dashed lines show the WT sample as calibrator. Error bars show the standard error of the mean. Multiple comparisons were made using one-way ANOVA, with significance levels: *P < 0.05; **P < 0.01; ***P < 0.001. Significant differences are indicated with symbols: # comparing to the induced WT strain (WT ATc), $ comparing to the induced control *M. smegmatis*:PLJR962 (PLJR962 ATc), * comparing to the respective uninduced strain. The • symbol indicates a highly significant difference (*P* < 0.0001) from all controls.

The normalized relative gene expression of the target genes *murT*, *gatD*, *namH* and neighboring genes was determined for selected samples, with and without inducer, at 6 h post-induction (**Figure 3**).

The obtained results show that the *murT* gene was successfully repressed in most induced *murT^-^* mutants (**Figure 3A**). A highly significant knockdown efficiency of *murT* was found with sgRNA1-*murT* comparing with WT ATc (53.5-fold; *P* < 0.0001), PLJR962 ATc (44.6-fold; *P* < 0.0001) or the respective uninduced mutant (57-fold; *P* < 0.0001). The same was observed with sgRNA3-*murT* (ranging from 16.5 to 21.1-fold repression) when compared to all controls (*P* < 0.0001). In contrast, sgRNA2-*murT* did not lead to a significant repression of *murT*. The highest repression of *murT* was achieved with sgRNA1-*murT*, being directly justified by PAM strength. Both sgRNA2-*murT* (PAM14, +1120 bp) and sgRNA3-*murT* (PAM15, +153 bp) have weak PAMs, however sgRNA3-mediated targeting resulted in a superior knockdown efficiency because sgRNA3-*murT* targets the beginning of the *murT* gene. An observable decrease in the mRNA expression of *gatD* was observed in all induced *gatD* ^-^ mutants (**Figure 3A**). The employed sgRNAs caused a fold repression ranging from 1.7 to 3.3 when compared with the respective uninduced mutants. A significant knockdown efficiency was found with sgRNA3-*gatD* (*P* = 0.0103, comparing with the correspondent uninduced mutant) and sgRNA4-*gatD* (*P* = 0.0433, comparing with WT ATc). CRISPRi-mediated silencing of the *murT* and *gatD* genes did not cause any significant polar effects in neighboring genes (**Figures 3** and **S3**).

Furthermore, the *namH* gene was also successfully repressed in the induced *namH* ^-^ mutants (**Figure 3B**). A highly significant knockdown efficiency of *namH* was observed with sgRNA1-*namH* comparing with WT ATc (7.3-fold; *P* < 0.0001), PLJR962 ATc (10.1-fold; *P* < 0.0001) or the respective uninduced mutant (7.5-fold; *P* < 0.0001). Likewise, highly significant repression differences were also observed with sgRNA2-*namH* (ranging from 5.6 to 10.5-fold) and sgRNA3-*namH* (ranging from 5.3 to 8.3 - fold) when compared to all controls (*P* ≤ 0.0003). The highest repression efficiency was achieved with sgRNA1-*namH*, which targets the promotor region of the *namH* gene, therefore inhibiting transcription initiation (Qi et al., 2013). The repression of *namH* was also more efficient with sgRNA2-*namH* (PAM1, +318 bp) than with sgRNA3-*namH* (PAM4, +1142 bp) due to higher PAM strength and to the target site. The knockdown efficiency between these sgRNAs is similar because the fold repression discrepancy between strong PAMs is minimal (Rock et al., 2017). The lack of reverse polarity effects contrasts with the occurrence of significant downstream polar effects in *MSMEG_6411* with all sgRNAs (**Figure 3B**).

### 3.2 Construction of the double and triple mutants

To uncover synergistic effects between the action of beta-lactams and the depletion of the *N*-glycolylation of muramic acid and/or the D-*iso*-Glu amidation of PG, knockdown mutants were also constructed in previously modified strains, lacking BlaS, PM965 (*M. smegmatis* Δ*blaS1*) and PM979 (*M. smegmatis* Δ*blaS1* Δ*namH*) (Raymond et al., 2005).

For the construction of the double mutants, having PM965 as a parental strain, the above knockdown mutants of *murT*, *gatD* and *namH* were considered. *M. smegmatis* PM965 was transformed with the following sgRNA-containing CRISPRi plasmids targeting the genes of interest: sgRNA1 or sgRNA2 for *murT*, sgRNA2 or sgRNA4 for *gatD* and, sgRNA1 or sgRNA3 for *namH*. To select mutants with medium-high repression *vs*. medium-low repression, the plasmids were chosen based on theoretical assumptions (differences in PAM strength and target site) (Choudhary et al., 2015; Rock et al., 2017) and on the spotting phenotypes of the single mutants (**Figure 2**).

To facilitate the identification of synergies between the depletion of NamH and the MurT/GatD enzymatic complex, the triple mutants, using PM979 as a parental strain, were constructed. In this case, only the *murT* and *gatD* knockdown mutants were considered since PM979 already lacks the *namH* gene. The plasmids used for target repression were the same that were used for the double mutants.

#### 3.2.1 Phenotyping of the double and triple mutants through spotting assays

To phenotypically characterize the double and triple mutants, spotting assays were performed.

**Figure 4:**
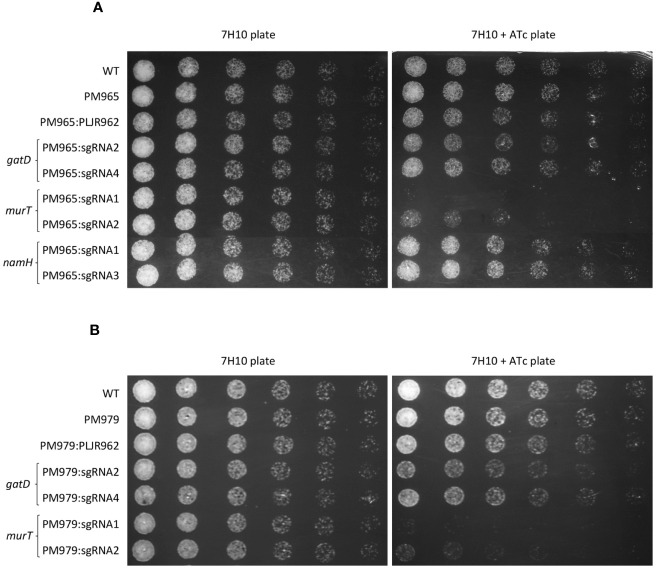
Spotting assays of *M. smegmatis* double and triple mutants (n=3): **(A)** double mutants, having PM965 (Δ*blaS1*) as a parental strain and one target gene repressed; **(B)** triple mutants, having PM979 (Δ*blaS1*Δ*namH*) as a parental strain and either *murT* or *gatD* repressed. Negative and positive controls were included in all experiments. The first spots were normalized to an OD_600_ of 0.001 and the next were performed by preparing and spotting 2-fold serial dilutions of each culture on agar plates.

The spotting assays of the double mutants (**Figure 4A**) revealed considerable growth defects for the induced Δ*blaS1 murT* ^-^ mutants, similar to those observed with the induced *murT* ^-^ mutants, with lower viability for sgRNA1-*murT* (PAM5, +406 bp) compared to sgRNA2-*murT* (PAM14, +1120bp). The phenotype of the induced Δ*blaS1 gatD* ^-^ mutants was also identical to that observed for the induced *gatD^-^* mutants, with slightly more severe growth differences with sgRNA2-*gatD* (PAM9, +299 bp) than with sgRNA4-*gatD* (PAM13, +652 bp). Moreover, the phenotype of the induced Δ*blaS1 namH* ^-^ mutants was indistinguishable from that observed for the induced *namH* ^-^ mutants.

The spotting assays of the triple mutants (**Figure 4B**) depicted slight changes in the viability of the induced Δ*blaS1* Δ*namH murT* ^-^ and Δ*blaS1* Δ*namH gatD* ^-^ mutants when compared to the double mutants. These minimal viability changes were mainly observed with the induced PM979:sgRNA2-*gatD* and Δ*blaS1* Δ*namH murT* ^-^ mutants.

#### 3.2.2 Assessment of CRISPRi-mediated gene knockdown by qRT-PCR

**Figure 5:**
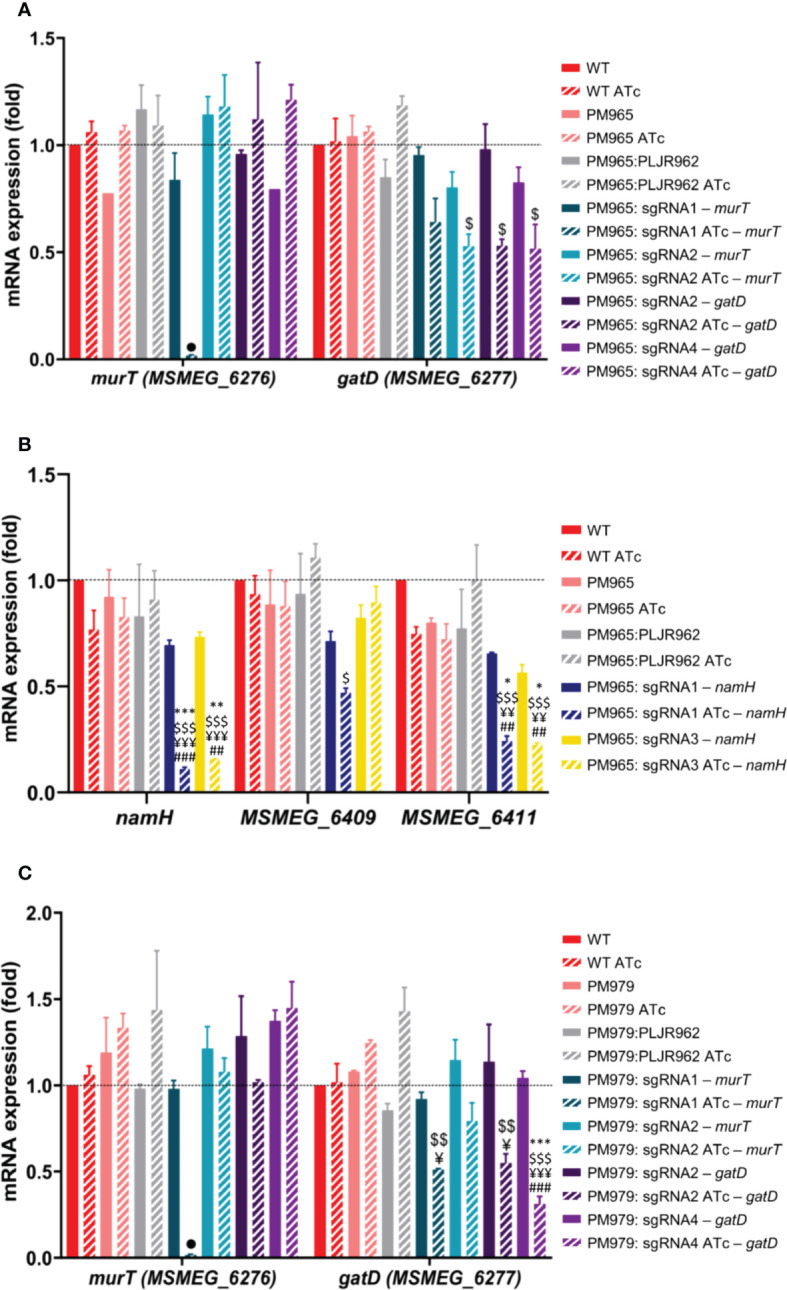
Results of qRT-PCR assays for the double and triple mutants (n=2): **(A, B)** Double mutants: **(A)** *murT* (blue) and *gatD* (purple) knockdown mutants constructed in PM965; **(B)** *namH* knockdown mutants constructed in PM965; **(C)** Triple mutants: *murT* (blue) and *gatD* (purple) knockdown mutants constructed in PM979. The graph shows the mean of the relative mRNA expression of genes of interest normalized to *sigA*, at 6 hours post-induction, with (stripped bars) and without (smooth bars) ATc. Dashed lines show the WT as calibrator. Error bars show the standard error of the mean. Multiple comparisons were made using one-way ANOVA, *P < 0.05; **P < 0.01; ***P < 0.001. Significant differences are indicated with symbols: # comparing to control *M. smegmatis* WT ATc, ¥ comparing to controls PM965 ATc or PM979 ATc, $ comparing to PM965:PLJR962 ATc or PM979:PLJR962 ATc, * comparing to the respective uninduced strain. The • symbol indicates a highly significant difference (*P* < 0.0001) from all controls.

Similarly, to the single mutants, the normalized relative gene expression of genes *murT*, *gatD*, *namH* and neighboring genes was also evaluated for the double and triple mutants in induced and non-induced conditions (**Figure 5**).

In the case of the induced Δ*blaS1 murT* ^-^ mutants (**Figure 5A**), sgRNA1-*murT* (PAM5, +406 bp) led to a highly significant repression of *murT* when compared to WT ATc (64.1-fold; *P* < 0.0001), PM965 ATc (64.6-fold; *P* < 0.0001), PM965:PLJR962 ATc (66-fold; *P* < 0.0001) or the respective uninduced mutant (50.6-fold; *P* < 0.0001). In contrast, sgRNA2-*murT* (PAM14, +1120bp) did not lead to a significant repression of *murT*. Significant polar effects in the mRNA expression of *gatD* were only observed with sgRNA2-*murT* when compared to PM965:PLJR962 ATc (2.2-fold; *P* = 0.0337). In the case of the Δ*blaS1 gatD* ^-^ mutants (**Figure 5A**), a significant repression of *gatD* was attained with both sgRNA2-*gatD* (2.2-fold; *P* = 0.0373) and sgRNA4-*gatD* (2.3-fold; *P* = 0.019) when compared to control strain PM965:PLJR962 ATc. Following the repression of either *murT*/*gatD*, no upstream or downstream polar effects were found in neighboring genes (**Figures 5** and **S4**).

In the case of the induced Δ*blaS1 namH* ^-^ mutants (**Figure 5B**), a highly significant repression of *namH* was obtained with both sgRNA1-*namH* (ranging from 6.3 to 8.2-fold) and sgRNA3-*namH* (ranging from 4.7 to 5.8-fold repression), when compared to all controls (*P* ≤ 0.0017). A higher knockdown efficiency was obtained with sgRNA1-*namH* (PAM11, -44bp) than with sgRNA3-*namH* (PAM4, +1142 bp) as sgRNA1-*namH* targets the promoter region and possesses a higher PAM strength. Similarly, to the single mutants, significant downstream polar effects in *MSMEG_6411* were observed with both sgRNAs. In terms of reverse polarity, sgRNA1-*namH* caused a significant decrease in the mRNA expression levels of *MSMEG_6409* when compared to PM965:PLJR962 ATc (2.4-fold; *P* = 0.0322). This might have occurred due to an overlap between the target region and the promoter guiding the transcription of *MSMEG_6409*, since sgRNA1-*namH* targets the promoter region of *namH* and *MSMEG_6409* is transcribed in the antisense strand (**Figure 1B**).

Again, a highly significant repression of *murT* was achieved with sgRNA1-*murT* (ranging from 56.2 to 82.5–fold repression) when compared to all controls (*P* < 0.0001), in the case of the induced Δ*blaS1* Δ*namH murT* ^-^ mutants (**Figure 5C**). Contrary to what was observed for the double mutants, significant polar effects in the mRNA levels of *gatD* were observed with sgRNA1-*murT* when compared to PM979 ATc (2.4-fold; *P* = 0.0113) and PM979:PLJR962 ATc (2.8-fold; *P* = 0.0034). In the case of the induced Δ*blaS1* Δ*namH gatD* ^-^ mutants, *gatD* was significantly repressed with sgRNA2-*gatD* when compared to PM979 ATc (2.3-fold; *P* = 0.0211) and to PM979:PLJR962 ATc (2.6-fold; *P* = 0.0062) (**Figure 5C**). A highly significant repression of *gatD* (ranging from 3.3 to 4.6–fold) was also achieved with sgRNA4-*gatD*, comparing to all controls (*P* ≤ 0.0009). No significant polar effects were found in neighboring genes following the repression of the *murT*/*gatD* operon (**Figure S5**).

Since the triple mutants have *M. smegmatis* PM979 as a parental strain (Δ*blaS1* Δ*namH*), the mRNA levels of *namH* were not evaluated.

### 3.3 Minimum inhibitory concentration determination of the constructed mutants

**Figure 6:**
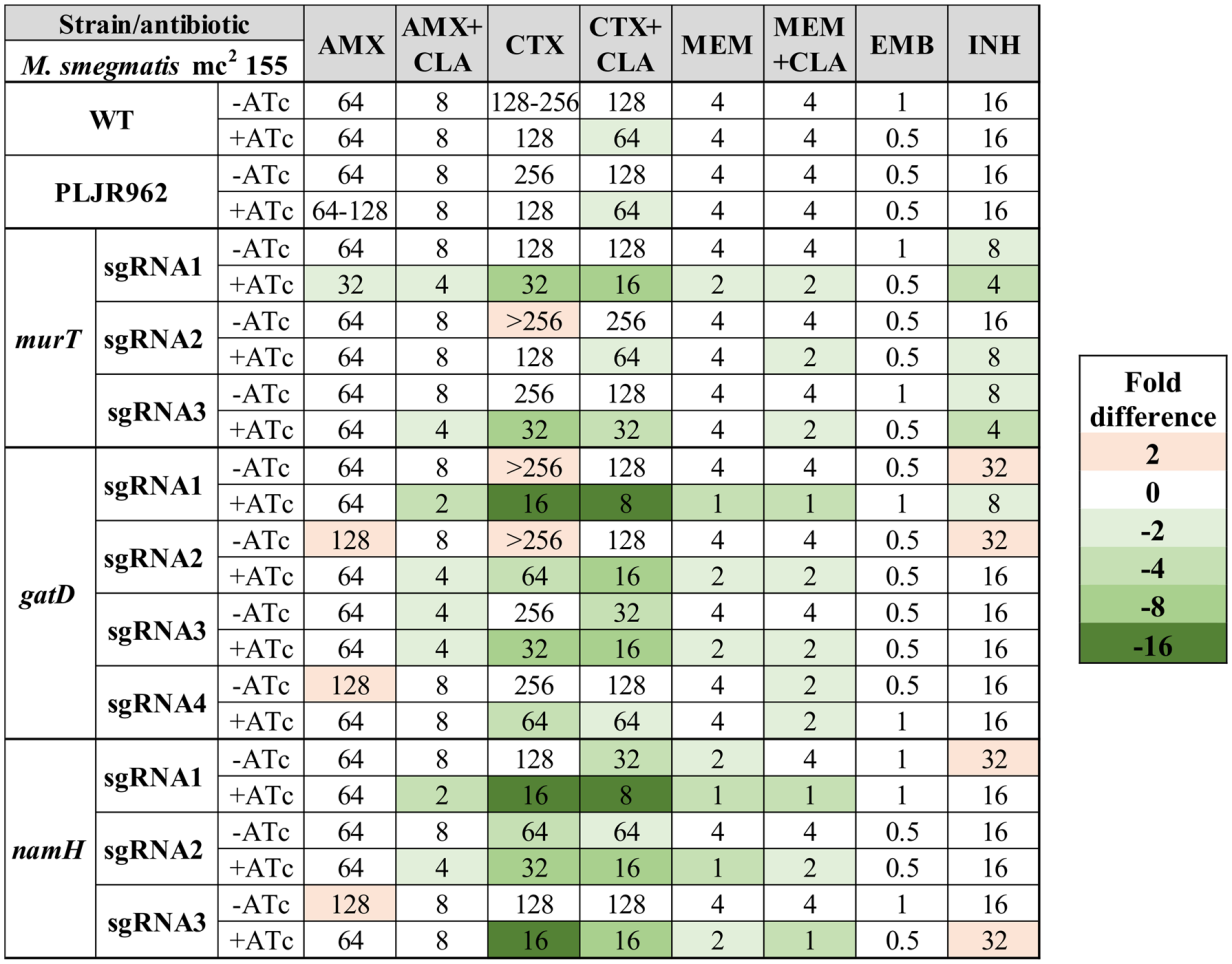
Heatmap of the fold differences in minimum inhibitory concentrations (MICs) of the knockdown mutants constructed in the *M. smegmatis* WT strain, with and without ATc. Columns represent the antibiotics and rows represent the strains. The median MIC (in µg/mL) of each antibiotic for each strain is shown in the respective intersection and colours depict an increase (red shades) or decrease (green shades) in the value when compared to the MICs of *M. smegmatis* WT. AMX, amoxicillin; CLA, clavulanate; CTX, cefotaxime; EMB, ethambutol; INH, isoniazid; MEM, meropenem.

To investigate the role of the D-*iso*-Glu amidation and of the *N*-glycolylation of PG in antibiotic susceptibility, the MICs of ethambutol, isoniazid and three beta-lactams, with or without clavulanate, were determined against the constructed mutants and respective control strains (**Figure 6**). For these experiments, 100 ng/mL of ATc were used since the MIC of ATc against *M. smegmatis* mc^2^ 155 WT has been defined as 500 ng/mL (Ehrt et al., 2005). Moreover, other studies have also used 100 ng/mL of ATc to induce CRISPRi-mediated silencing in *M. smegmatis* WT (Singh et al., 2016; Rock et al., 2017), including those employing the dCas9_Sth1_-based system (Rock et al., 2017).

As expected, the observed MICs for the negative controls (*M. smegmatis* WT, *M. smegmatis*:PLJR962) were not considerably influenced by the addition of ATc (Marques, 2020; Silveiro, 2020). Notably, the repression of the *murT*/*gatD* operon and of the *namH* gene promoted increased susceptibility to all tested beta-lactams (Marques, 2020; Silveiro, 2020). The MICs of AMX remained mostly constant, as 2-fold differences can occur due to technical variability and are, thus, not considerable. Substantial susceptibility differences (≥ 4-fold) in AMX+CLA were found when *gatD* was repressed with sgRNA1-*gatD* or when *namH* was silenced with sgRNA1-*namH*. Hence, the correspondent induced knockdown mutants are susceptible to AMX+CLA according to the EUCAST breakpoints (**Table S4**). In the case of the induced *M. smegmatis*:PLJR962:sgRNA1-*gatD* knockdown mutant, the susceptibility gain might be explained by a viability loss, as shown in the phenotyping assays. Unlike CTX or MEM, the addition of CLA to beta-lactams caused marked decreases in the MIC of AMX. The most notable susceptibility differences (ranging from 4 to 16-fold) occurred with CTX (+CLA), being present across all induced knockdown mutants, independently of the target gene. The most extensive increases in susceptibility to CTX (+CLA) occurred when *gatD* was repressed with sgRNA1-*gatD* or, when *namH* was silenced with sgRNA1-*namH* and sgRNA3-*namH*. Additionally, the fitness loss of the induced *M. smegmatis*:PLJR962:sgRNA1-*gatD* knockdown mutant also provoked a 4-fold decrease in the MIC of MEM (+CLA). In contrast, the repression of *namH* consistently promoted increased susceptibility to MEM (+CLA), thereby inferring a particular role of NamH in resistance to the carbapenem. Indeed, all knockdown mutants with a meropenem MIC ≤ 2 µg/mL are classified as susceptible to MEM according to the breakpoints (**Table S4**). The MICs of EMB and INH remained essentially unchanged, with an interesting exception. The repression of *murT* with sgRNA1-*murT* and sgRNA3-*murT* caused a 4-fold decrease in the MIC of INH, which is most likely due to viability loss of the respective mutants.

**Figure 7:**
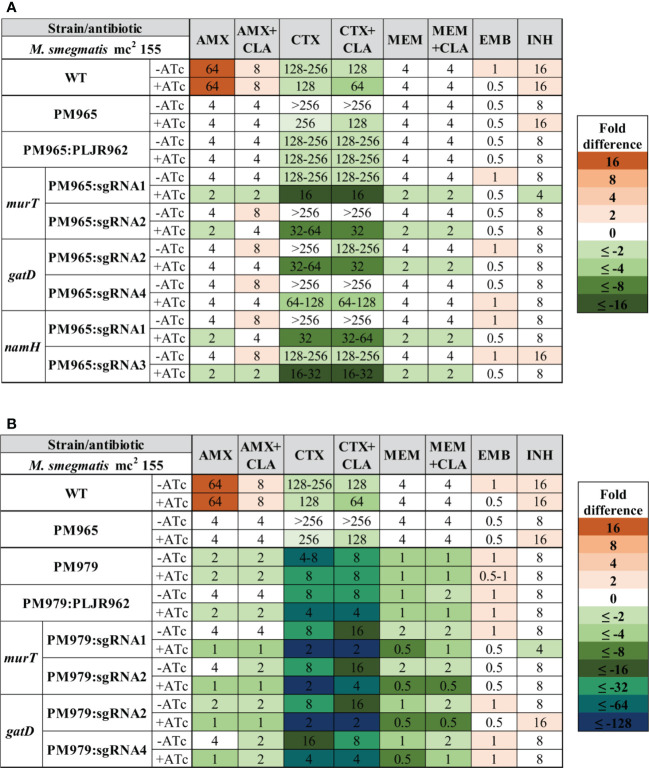
Heatmap of the fold differences in minimum inhibitory concentrations (MICs) of the double and triple knockdown mutants, with and without ATc. **(A)** Mutants constructed in *M. smegmatis* PM965 strain (double mutants); **(B)** Mutants constructed in the *M. smegmatis* PM979 strain (triple mutants). Columns represent the antibiotics and rows represent the strains. The median MIC (in µg/mL) of each antibiotic for each strain is shown in the respective intersection and colours depict an increase (red shades) or decrease (green/blue shades) in the value when compared to the MICs of control strain *M. smegmatis* PM965 (Δ*blaS1*). AMX, amoxicillin; CLA, clavulanate; CTX, cefotaxime; EMB, ethambutol; INH, isoniazid; MEM, meropenem.

Similarly, the median MICs of the double mutants were determined for the same antibiotics (**Figure 7A**). The MICs of *M. smegmatis* PM965 and PM965:PLJR962 remained unaltered with the addition of ATc. When compared to *M. smegmatis* WT, the parental PM965 strain presented substantially lower MICs to all beta-lactams because this strain lacks the major beta-lactamase of *M. smegmatis*, BlaS (Raymond et al., 2005). The absence of BlaS in conjunction with the repression of *murT*/*gatD* and *namH* promoted increased susceptibility to the tested beta-lactams, although with insubstantial differences in the MICs of AMX (+CLA) and MEM (+CLA). The lack of BlaS in PM965 aided the classification of the induced *murT* and *namH* knockdown mutants as susceptible to AMX, according to the breakpoints (**Table S4**). Both induced *M. smegmatis* PM965:PLJR962:sgRNA1-*murT* and PM965:PLJR962:sgRNA1-*namH* are classified as susceptible to AMX+CLA according to the breakpoints. The most notable susceptibility differences, ranging from 4 to 16-fold in comparison with *M. smegmatis* WT and PM965, occurred with CTX (+CLA), being present across most induced knockdown mutants. Extensive susceptibility differences (≥16-fold) from the PM965 strain in CTX (+CLA) were found when *murT* was repressed with sgRNA1-*murT* or when *namH* was silenced with sgRNA3-*namH*. The increased susceptibility of the induced *M. smegmatis* PM965:PLJR962:sgRNA1-*murT* knockdown mutant to beta-lactams might be explained by fitness loss, as shown in the phenotyping assays. All induced knockdown mutants constructed in the BlaS-depleted strain except for induced *M. smegmatis* PM965:PLJR962:sgRNA4-*gatD* are susceptible to MEM according to the breakpoints (**Table S4**). The MICs of EMB and INH remained essentially unchanged.

Similarly, the median MICs of the triple mutants, having *M. smegmatis* PM979 as a parental strain, were determined for the same antibiotics (**Figure 7B**). The MICs of *M. smegmatis* PM979 were not considerably altered by ATc addition. In fact, the PM979 strain was found to be very susceptible to beta-lactams when compared to the WT and PM965 strains, suggesting a major role for the *N*-glycolylation of muramic acid in resistance to beta-lactams. The MICs of AMX (+CLA), CTX (+CLA) and MEM+CLA against *M. smegmatis* PM979:PLJR962 suffered a 2-fold decrease when inducer was added, with the obtained MIC values for AMX (+CLA) and MEM+CLA being equal to those obtained with the PM979 strain in induced conditions. Therefore, the MIC differences observed for the knockdown mutants in CTX (+CLA) cannot be interpreted as a result of their knockdowns alone since these differ in 2-fold from the MIC values obtained with the induced PM979 strain. This reproducible effect is most likely due to dCas9_Sth1_-associated toxicity as an exogenous protein to the bacterial system. Despite this remark, the additional repression of *murT*/*gatD* in the PM979 strain favoured even lower beta-lactam MICs. The cumulative effect of the absence of BlaS and NamH and of the depletion of the D-*iso*-glutamate amidation of PG provoked increased susceptibility to beta-lactams when compared to the parental strain PM979 (2 or 4-fold differences), and especially when compared to *M. smegmatis* WT and PM965 (4 to 128-fold differences). Although *M. smegmatis* PM979 is already very susceptible to beta-lactams when compared to the WT and PM965 strains, the additional repression of *murT*/*gatD* favored even lower beta-lactam MICs. When compared to the PM965 strain, all sgRNAs leading to depleted D-*iso*-Glu amidation caused considerable 4-fold decreases in the MIC of AMX, with comparable results for AMX+CLA. Most sgRNAs caused a 64 to 128-fold reduction in the MIC of CTX (+CLA) compared to PM965 and WT MIC values. Moreover, a noteworthy MEM MIC value of 0.5 µg/mL was obtained with all sgRNAs, differing in 8-fold from the PM965 and WT MIC values. Although the fold differences in the MIC of MEM between the triple mutants and the PM965 control strain seem to be less substantial, the PM965 MIC value for MEM is extremely low in comparison to that of AMX and CTX, making it difficult to attain great fold differences. Furthermore, all induced knockdown mutants are susceptible to AMX (+CLA) and MEM according to the breakpoints (**Table S4**). Again, the MICs of EMB and INH remained essentially unchanged.

### 3.4 Macrophage infection and intracellular survival assays of the constructed mutants

**Figure 8:**
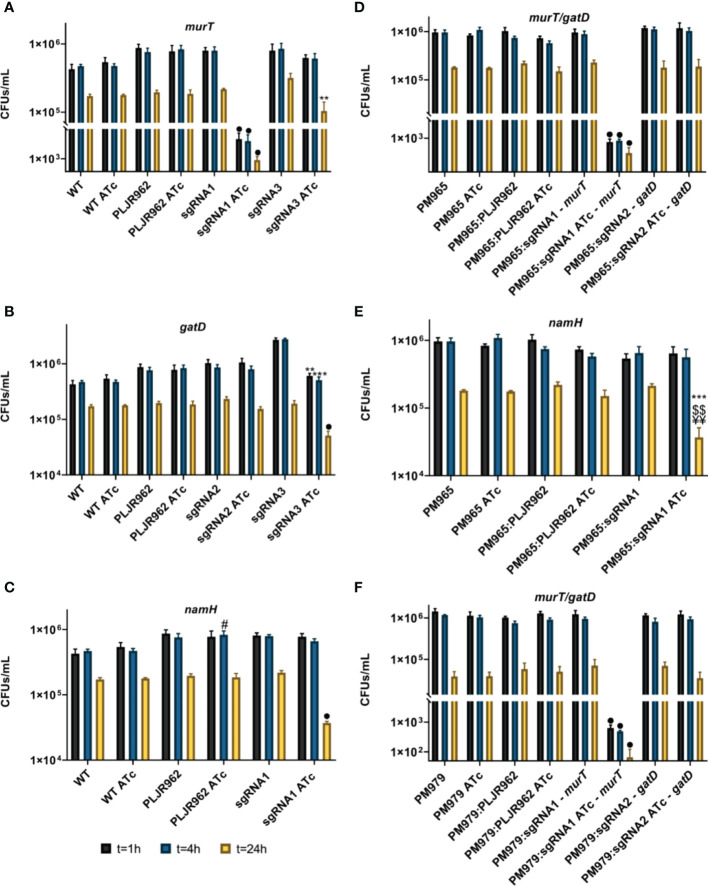
Logarithmic representation of the mean of bacterial survival, with/without ATc, in CFUs/mL, after infection and disruption of J774.A1 macrophages (n=3). **(A–C)** Single mutants: **(A)** *murT* knockdown mutants; **(B)** *gatD* knockdown mutants; **(C)** *namH* knockdown mutants; **(D, E)** Double mutants: **(D)** *murT*/*gatD* knockdown mutants; **(C)** *namH* knockdown mutants; **(F)** Triple mutants, *murT*/*gatD* knockdown mutants. Error bars show the standard error of the mean. Multiple comparisons were made using one-way ANOVA: *P < 0.05; **P < 0.01; ***P < 0.001. Significant differences are indicated with symbols: # comparing to control strain WT ATc, ¥ comparing to the controls PM965 ATc or PM979 ATc; $ comparing to *M. smegmatis* WT/PM965/PM979:PLJR962 ATc, * comparing to the respective uninduced strain. The • symbol indicates a highly significant difference (*P* < 0.0001) from all controls.

To elucidate the contribution of the D-*iso*-Glu amidation and of the *N*-glycolylation of PG to mycobacterial intracellular killing, J774 murine macrophages were infected with all strains at a MOI of 5 and, disrupted for the assessment of the intracellular survival of bacteria (in CFUs/mL) at 1 h, 4 h and 24 h post-infection (**Figure 8**).

As expected, the intracellular survival of mycobacteria decreased over time (Marques, 2020; Silveiro, 2020). The single mutants’ results show that the mean CFUs/mL of the negative controls *M. smegmatis* WT and PLJR962 were not significantly influenced by ATc addition (**Figures 8A-C**). The repression of *murT* with sgRNA1-*murT* caused a highly significant decrease in the intracellular survival of these bacteria at all timepoints (**Figure 8A**; *P* < 0.0001, when compared to *M. smegmatis* WT ATc, PLJR962 ATc or the respective uninduced control). This observation is explained by a strong repression of the *murT* gene, causing a lethality effect that can be verified by the low viability of the correspondent induced *murT* ^-^ mutant in the phenotyping assays. Besides, the repression of *murT* with sgRNA3-*murT* provoked a significant decrease in intracellular bacterial survival at t = 24 h (**Figure 8A**; *P* = 0.004, comparing with the respective uninduced control) while retaining some viability in the phenotyping assays. A highly significant decrease in the intracellular survival of the induced *gatD* knockdown mutants at t = 24 h was only attained with sgRNA3-*gatD* (**Figure 8B**; *P* < 0.0001, compared to all controls). Furthermore, the knockdown of *namH* attained with sgRNA1-*namH* also caused a highly significant decrease in the intracellular survival of bacteria at t = 24 h (**Figure 8C** **;** *P* < 0.0001, compared to all controls). These results indicate that both the D-*iso*-Glu amidation and the *N*-glycolylation of PG contribute to the intracellular survival of mycobacteria during infection. Moreover, the PM979 strain, lacking both BlaS and NamH, undergoes a significantly faster clearance by J774 macrophages when compared to WT or PM965 (**Figure S6**).

The mean CFUs/mL of the double (**Figures 8D, E**) and triple mutants (**Figure 8F**) were also determined. The controls of the double mutants (*M. smegmatis* PM965, *M. smegmatis* PM965:PLJR962) and of the triple mutants (*M. smegmatis* PM979, *M. smegmatis* PM979:PLJR962) did not display any differences in intracellular survival after the addition of inducer. Generally, the obtained results for the knockdown of *murT* and *gatD* in the PM965 and PM979 strains were identical to those observed with the single mutants (**Figure 8**). Unfortunately, the effect of D-*iso*-Glu amidation on mycobacterial intracellular survival could not be shown using the best candidate sgRNAs targeting *murT*/*gatD* leading to significant changes in the CFUs/mL because the respective mutants were not available. Still, the repression of *murT* in both PM965 and PM979 strains with sgRNA1-*murT* caused a highly significant decrease in the intracellular survival of these bacteria at all timepoints (*P* < 0.0001, compared to all controls) (**Figures 8D, F**). Remarkably, the knockdown of *namH* in the PM965 strain with sgRNA1-*namH* caused a very significant decrease in the bacterial intracellular survival at t = 24 h when compared to PM965 ATc (*P* = 0.0018), PM965:PLJR962 ATc (*P* = 0.0048) or the respective uninduced mutant (*P* = 0.0008) (**Figure 8E**).

### 3.5 Single nucleotide variants associated with differential susceptibility to beta-lactams in clinical strains of *Mtb*

To uncover whether the genes encoding the proteins responsible for the characteristic modifications of mycobacterial PG are of conserved nature, we benefited from the data obtained for 172 *Mtb* isolates in a previous study (Olivença et al., 2022b), where the susceptibility of the strains to AMX and MEM, with and without clavulanate, was also evaluated.

**Figure 9:**
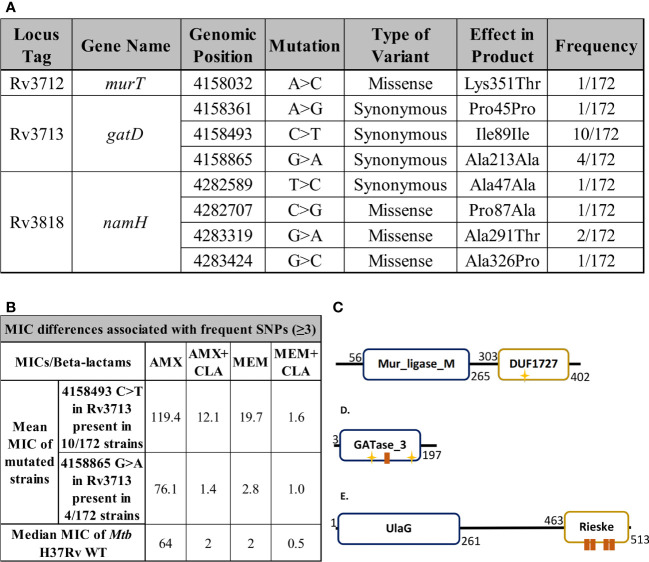
Single nucleotide polymorphism (SNP) detection and MIC assays. **(A)** Single nucleotide polymorphisms found in the target genes *murT*, *gatD* or *namH* through the whole genome sequencing of 172 clinical strains of *Mtb*. Locus tags, gene names, products and positions refer to the H37Rv reference genome (AL123456.3). **(B)** Geometric mean MIC of clinical strains harbouring frequent single nucleotide polymorphisms (n≥3) compared to the median MIC of *Mtb* H37Rv WT. **(C-E)** Protein structures were drawn based on data from the NCBI, Pfam, UniProt and PROSITE databases. The yellow stars represent active sites while the brown boxes represent binding sites. **(C)** The protein structure of MurT, comprising a central Mur ligase domain and the DUF1727 domain. The D349 residue present in the DUF1727 domain of MurT is important for the catalytic activity of GatD, being part of its catalytic triad. **(D)** The protein structure of GatD, comprising a GATase_3 domain, responsible for its catalytic glutamine amidotransferase activity (C94, H189). **(E)** The protein structure of NamH, comprising a beta-lactamase superfamily UlaG domain and a Rieske domain, associated with oxidoreductase reactions.

By performing a SNP-based analysis against the H37Rv reference genome, we found the *murT*, *gatD* and *namH* genes to be highly conserved in this specific set of clinical strains of *Mtb* (**Figure 9A**, **Tables S5**, **S6**). When a certain SNP was detected in three or more strains, the geometric mean of the MIC of these strains to AMX and MEM was compared to the median MIC of the control strain *Mtb* H37Rv WT (**Figure 9B**). Among the 172 strains, only one SNP (4158032 snp A>C) was identified for the *murT* gene, occurring in the region coding for the C-terminal domain of MurT (DUF1727) (**Figure 9C**). Although synonymous mutations do not carry as much importance as missense variants or indels, three synonymous variants were found in *gatD*. One of these mutations (4158493 snp C>T) was found in 10/172 strains, all of which were classified as lineage 2 strains and had increased beta-lactam MIC values, namely in the case of AMX+CLA and MEM (**Figure 9B**). This mutation occurred in the GATase_3 domain of GatD, near its first catalytic site (C94) (**Figure 9D**). Another one of these mutations (4158865 snp G>A) was found in only 4/172 strains, all of which were classified as lineage 4 strains, however without any considerable MIC differences (**Figure 9B**). Four variants were found for the *namH* gene, but always at a low frequency so they could not be associated with MIC changes. Two of these mutations (4282589 snp T>C; 4282707 snp C>G) occurred in regions coding for the UlaG domain of NamH whereas the other two (4283319 snp G>A; 4283424 snp G>C) occurred in regions coding for the Rieske domain (**Figure 9E**).

## 4 Discussion

The lack of treatment success of drug-resistant TB urges the development of novel antituberculosis agents (Singh and Chibale, 2021). The PG layer of the mycobacterial CW features unique modifications, such as the *N*-glycolylation of muramic acid and the amidation of D-*iso*-glutamate (Catalão et al., 2019; Maitra et al., 2019). In this study, these PG modifications were depleted to address their role in beta-lactam susceptibility and in intracellular survival. Given the emergence of MDR-TB and the necessity of novel TB therapeutic regimens, research on the introduction of beta-lactams in TB-therapy is considered important by the WHO (WHO, 2020). Our results, discussed below, support the development of therapeutic agents targeting the mycobacterial PG and suggest that the inhibition of PG polymerization could synergize with beta-lactams to aid the treatment of MDR-TB infections.

The phenotyping assays of the single PG modification knockdown mutants uncovered the essentiality of the *murT*/*gatD* operon for the viability of *M. smegmatis*, as reported by studies with the correspondent *Mtb* orthologues, *Rv3712*/*Rv3713* (Sassetti et al., 2003; Griffin et al., 2011; DeJesus et al., 2017; Maitra et al., 2021). In contrast, the muramic acid hydroxylase NamH, comprised of the N-terminal UlaG and the C-terminal Rieske domains, was found to be non-essential to the survival of *M. smegmatis*, also being in line with previous findings in *M. smegmatis* (Raymond et al., 2005) and in its *Mtb* orthologue, *Rv3818* (Sassetti et al., 2003; Griffin et al., 2011; DeJesus et al., 2017). Overall, the obtained growth defects for sgRNAs with varying PAM strength usually matched the anticipated phenotypes whilst revealing a direct correlation between these and the sgRNA targeting site distance from the TSS.

The qRT-PCR results confirmed the successful repression of all target genes, however with variable efficiency. The repression of *gatD* had an extremely low efficiency when compared to that of *murT*, possibly due to differences in target vulnerability (Bosch et al., 2021). The repression efficiency of *murT* was also found to be higher than that of *namH*, which could be explained by the fact that sgRNAs targeting essential genes usually lead to a greater knockdown efficiency than sgRNAs targeting non-essential genes (Bosch et al., 2021). In few cases, a reduction in the mRNA levels of the target genes was observed even without inducer, indicating leaky expression. Moreover, an indirect proportionality between the efficiency of repression and the distance between target site and the TSS was uncovered. Although researchers were unable to observe any link between repression efficiency and target site using the same CRISPRi system (Rock et al., 2017; Wong and Rock, 2021), a previous study using a CRISPRi system based on the use of dCas9 from *Streptococcus pyogenes* (dCas9_Spy_) is concordant with our results (Choudhary et al., 2015). Despite using the same CRISPRi system, it is noteworthy that the obtained levels of knockdown are not as robust as those previously described for the repression of several target genes in *M. smegmatis* (11–226 fold) (Rock et al., 2017). An induction time of 6 h was used, which is low in comparison to the 14 h of induction previously reported (Rock et al., 2017). Nevertheless, the CRISPRi system reaches maximal silencing efficiency between one and two cell divisions after ATc addition (Rock et al., 2017), meaning that 6 h of induction time should be sufficient to assess the depletion of target mRNAs. Besides, knockdown efficiency would likely be improved by predepletion in ATc-supplemented medium. Overall, our results show that dCas9_Sth1_-mediated targeting leads to efficient target inhibition, thus affecting the associated phenotypes.

The double and triple mutants, lacking BlaS, facilitate the discovery of synergistic effects between the action of beta-lactams and the depletion of characteristic PG modifications. The double mutants (Δ*blaS1 murT^-^*, Δ*blaS1 gatD^-^*, Δ*blaS1 namH* ^-^) presented a phenotype similar to that of the single knockdown mutants. Likewise, the simultaneous depletion of NamH and MurT/GatD (Δ*blaS1 ΔnamH murT^-^* and Δ*blaS1 ΔnamH gatD^-^* mutants) caused minimal changes in viability. The obtained knockdown efficiency of *murT*/*gatD* and *namH* was generally comparable between the single, double, and triple mutants, thereby suggesting a successful repression of all target genes independently of the parental strain. Again, an indirect proportionality between repression efficiency and the distance between the target site and the TSS was observed.

The median MICs obtained for the single mutants showed that the depletion of the *N*-glycolylation and the D-*iso*-Glu amidation of PG promote increased susceptibility to beta-lactams. Previous studies with *S. aureus* have also identified the MurT/GatD enzymatic complex as a mechanism of resistance to beta-lactams (Figueiredo et al., 2012; Münch et al., 2012). As expected, the obtained results revealed a direct proportionality between decreases in the MIC and the overall strength of the employed sgRNA. The cumulative absence of BlaS in conjunction with the knockdown of either *namH* or *murT*/*gatD* (double mutants) resulted in increased susceptibility to all beta-lactams. In this case, the most notable susceptibility differences pertaining to WT MICs (16 to 32- fold) occurred with AMX since BlaS is thought to possess substantially superior catalytic affinity for penicillins, when compared to cephalosporins and carbapenems (Solapure et al., 2013). The MIC results of the triple mutants demonstrated that repressing *namH* is a good strategy to increase susceptibility to beta-lactams. This could be explained by the role that *N*-glycolylated muropeptides play in strengthening CW stability through hydrogen bonds or by a differential recognition of the *N*-glycolylated glycan backbone by transpeptidases (Raymond et al., 2005; Silveiro, 2020). Together, the simultaneous depletion of the *N*-glycolylation of muramic acid and of the amidation of D-*iso*-Glu seem synergistic in such a way that these mutants present further reduced MIC values. Therefore, the development of a novel anti-TB drug that simultaneously targets the NamH and MurT/GatD proteins would be particularly effective.

Throughout these assays, MEM consistently produced the lowest MIC values when compared to other beta-lactams. While MEM inhibited mycobacterial growth very efficiently, CTX did not. To inhibit PG biosynthesis and kill mycobacteria, all sort of transpeptidases need to be inhibited (Kieser et al., 2015; Story-Roller and Lamichhane, 2018). Both MEM and CTX can inhibit PBPs and Ldts (Dubée et al., 2012) but only MEM can do so efficiently while being an extremely poor substrate to beta-lactamases (Tremblay et al., 2010). This observation supports the recent efforts to repurpose meropenem-clavulanate to treat MDR-TB. Moreover, we found that some reductions in beta-lactam MICs were associated with a loss of viability of the respective induced knockdown mutants, namely when either *murT* or *gatD* were targeted. Although not as markedly, this increase in susceptibility to beta-lactams was also seen when weaker sgRNAs were used to target these genes. Furthermore, the most notable susceptibility differences occurred with CTX (+CLA), being present across most induced knockdown mutants. Besides inhibiting PBPs, CTX can also inhibit some Ldts (Dubée et al., 2012), the most active transpeptidases during PG cross-linking in mycobacteria (Lavollay et al., 2008; Kumar et al., 2012; Baranowski et al., 2018). Furthermore, the MurT/GatD enzymatic complex regulates the activity of Ldts in *Mtb*, thereby determining the levels of PG cross-linking (Pidgeon et al., 2019). Therefore, our observation could be explained by a favorable use of D-*iso*-Glu amidated PG as a substrate by transpeptidases that are targeted by CTX.

The immune recognition of mycobacterial PG by macrophages occurs through specific immune receptors, the cytosolic Nod-like receptors (NLRs) (Coulombe et al., 2009; Hansen et al., 2014), possibly justifying a more bactericidal response of macrophages. Whereas the recognition of molecules containing D-Glu-*m*-DAP is achieved by NOD1, PG–derived muramyl dipeptide (MDP) is mostly recognized by NOD2 (Coulombe et al., 2009; Hansen et al., 2014). NOD2-mediated host responses (NMHR) culminate in the production of pro-inflammatory cytokines possibly leading to oxidative burst responses (Coulombe et al., 2009; Hansen et al., 2014). Our intracellular survival assays showed that the depletion of MurT/GatD as well as that of NamH are both associated with an increased clearance rate, possibly due to a higher recognition by host macrophages.

The amidation of D-*iso*-Glu has been reported to lead to reduced immune recognition by NOD1 (Wang et al., 2016), thereby being an immune evasion strategy that enables mycobacteria to survive inside host macrophages during TB infection (Marques, 2020), being in conformity with our results.

On the other hand, previous studies revealed that *N*-glycolylated muropeptides are more stimulatory than *N*-acetylated muropeptides at activating NMHR (Coulombe et al., 2009). Concurring with these observations, other researchers have observed the same while reporting no differences in CFUs/mL between *Mtb* WT and Δ*namH* strains (Hansen et al., 2014). These observations imply that the *N*-glycolylation of muramic acid is associated with higher immune recognition, which could lead to decreased intracellular survival. However, our results are not concordant with these observations, perhaps because our infection assays were performed with saprophytic *M. smegmatis* instead of pathogenic *Mtb*. Nevertheless, our results showed that the *N*-glycolylation of PG clearly contributes to the increased intracellular survival of *M. smegmatis* in J774 macrophages. In accordance with our results, a previous publication demonstrated that *N*-glycolylated muropeptides provoke a reduced NOD2 activation when compared to their *N*-acetylated counterparts (Wang et al., 2016). Therefore, the role of NamH in the context of immune recognition and host-pathogen interactions remains unclear.

Our results also demonstrated that the triple mutants underwent a faster clearance by J774 macrophages when compared to the single or double mutants. Hence, simultaneously depleting the activity of NamH and of the MurT/GatD protein complex might be a good strategy to facilitate bacilli clearance during TB infection, further underlining the potential of these proteins as novel anti-TB therapeutic targets.

The high prevalence of antibiotic resistance in the TB-therapy setting urges the discovery of conserved drug targets, that is, targets which do not undergo mutation due to the selective pressure associated with the current TB therapeutic schemes. By analyzing the largest dataset of whole genome sequencing data and associated beta-lactam MICs against clinical strains of *Mtb* to date, our results have found *murT*, *gatD* and *namH* to be highly conserved, implying crucial and unique roles of these genes in the fitness of mycobacteria. Despite being often overlooked, synonymous mutations can lead to changes in the modulation of transcription and translation, thereby resulting in altered protein levels (Bailey et al., 2021). We uncovered one synonymous mutation in *gatD* (4158493 snp C>T) associated with increased resistance to AMX+CLA and MEM in 10/172 strains. Our results are in conformity with previous reports (Tsolaki et al., 2004; Maitra et al., 2021) and further emphasize the potential of MurT/GatD and NamH as novel anti-TB targets.

Our work is not without limitations. First, CRISPRi is known to cause either reverse or downstream polarity effects in operonic genes (Qi et al., 2013; Choudhary et al., 2015). However, the fact that operonic genes are usually co-transcribed, performing similar molecular functions, somewhat alleviates this problem (Singh et al., 2016; Rock et al., 2017; Bosch et al., 2021). Moreover, the double and triple mutants were constructed in previously modified strains based on theoretical assumptions. Furthermore, the sample of *Mtb* clinical strains used for the detection of SNPs associated with differences in drug susceptibility involved the overrepresentation of certain phylogenetic lineages in detriment of others (Olivença et al., 2022b). Although we advocate for the development of small molecules able to inhibit these potential antitubercular targets, we are aware that small molecules are not as effective as CRISPRi at inhibiting all enzyme functions (Bosch et al., 2021). At last, substantial differences between *M. smegmatis* and the pathogen exist and inferred conclusions with the surrogate strain may sometimes not apply to *Mtb*.

To the best of our knowledge, this is the first study that attempted to simultaneously deplete the activity of MurT/GatD and NamH and phenotypically characterize their conjoint relevance in the modulation of beta-lactam susceptibility and of host-pathogen interactions in mycobacteria. In conclusion, the characteristic modifications of mycobacterial PG were found to promote antibiotic resistance and intracellular survival inside host macrophages, contributing to limited antimicrobial effects and immune evasion. Inhibiting these PG modifications, whether individually or simultaneously, may arise as a strategy to fight TB disease and reduce the duration of TB therapeutic regimens in the future.

## Data availability statement

The datasets presented in this study can be found in online repositories. The names of the repository/repositories and accession number(s) can be found in the article/**Supplementary Material**.

## Author contributions

CS, MM and MJC conceived and designed the study. CS, MM, DM, and FO performed the experiments. CS, MM, FO, DP, AN, MP, EA and MC analyzed the data. CS wrote the original draft. All authors contributed to the article and approved the submitted version.

## Footnotes

1
https://primer3.ut.ee/

2
https://www.eucast.org/clinical_breakpoints/
