# Supplementary material for: CRISPRi-mediated characterization of novel anti-tuberculosis targets: Mycobacterial peptidoglycan modifications promote beta-lactam resistance and intracellular survival
Source: Front Cell Infect Microbiol. 2023 Mar 15;13:1089911. doi: 10.3389/fcimb.2023.1089911 (PMC10050696; doi:10.3389/fcimb.2023.1089911)
Supplement: Supplementary file 1 [file DataSheet_1.pdf]

frontiers

Supplementary Material

1 Supplementary Figures and Tables

1.1 Supplementary Figures

```
CLUSTAL O(1.2.4) multiple sequence alignment

sp|A0A0H3JUU7|MURT_STAAN      MRQWTAIHLAKLARKA---SRAVG--KRGTDLPGQIA  32
sp|Q8DNZ9|MURT_STRR6          MNLKTT--LGLLAGRSSHFVLSRL--GRGSTLPGKVA  33
tr|A0R5Q7|A0R5Q7_MYCS2        -MLTVRGRAALAAGAAARWASRVTGRGAGAMIGGLVA  36
tr|I6Y4C7|I6Y4C7_MYCTU        -MVTTRARLALAAGAGARWASRVTGRGAGAMIGGLVA  36
                                  .    .  *  .           *: : * :*

RKVDTDVLRKLAEQVDDIVFISGTNGKTTTSNLIGHTLKANNIQIIHNNEGANMAAGITSAFIMQST  99
LQFDKDILQSLAKNY-EIVVVTGTNGKTLTTALTVGILKEVYGQVLTNPSGANMITGIATTFLTAKS  99
MTLDRSVLRQLGQGR-RTAIVTGTNGKSTTTRMIAAALA-PLGPVASNTEGANMDAGLVSALAANR-  100
MTLDRSILRQLGMGR-RTVVVTGTNGKSTTTRMTAAALG-TLGAVATNAEGANMDAGLVAALAAHR-  100
  .* .:*:.*.      ..::*****: *: :    *      :  * .**** :*:.:::

P--KTKIAVIEIDEGSIPRVLKEVTPSMMVFTNFFRDQMDRFGEIDIMVNNIAETISNKG-IKLLLN  163
SKTGKNIAVLEIDEASLSRICDYIQPSLFVITNIFRDQMDRFGEIYTTYNMILDAIRKVPTATVLLN  166
---DAGLAALEVDEMHVPHVSDAVSPSVIVLLNLSRDQLDRVGEINHIERTLRAGLARHPDAVIVAN  164
---DAELAVLEVDEMHVPHISDAVDPAVVVLLNLSRDQLDRVGEINVIERTLRAGLARHPDAVVVAN  164
      :*.:*:**  : :: . : *::.*: *: ***:**.***    . :   : .     :: *

ADDPFVSRLKIASDTIVYYGMKAHAHEFEQ-STMNESRYCPNCGRLLQYDYIHYNQIGHYHCQ-CGF  228
GDSPLFYKPTI-PNPIEYFGFDLEKGPAQLAHYNTEGILCPDCQGILKYEHNTYANLGAYICEGCGC  232
CDDVLMTSAAYDNPDVVWVA--------AGGTWANDSVSCPRSGEVIVRD------GRDWYSTGTDF  217
CDDVLMTSAAYDSPNVVWVA--------AGGAWSNDSVSCPRSGEVIVRKAP--SQEDHWYSTGADF  221
 *. :.         : : .              .:.  ** .  ::  .         : .   .

KREQAKYEISSFDVA--PFLYLNINDEKYDMKIAGDFNAYNALAAYTVLRELGLNEQTIKNGFETYT  293
KRPDLDYRLTKLVELTNNRSRFVIDGQEYGIQIGGLYNIYNALAAVAIARFLGADSQLIKQGFDKSR  299
KRPSPQWWFDETHIHGPDGLS-----VPMELALPGTVNRGNATQAVAAAVALGADPVAAVAAVSTVD  279
KRPAPHWWFDDATLYGPDGLA-----LPMRLALPGSVNRGNAAQAVAAAVALGADPAVAVAAVCQVD  283
**   .: : .                   : : *  *  ** * :    ** :      ..

                       I                             II
SDNGRMQYFKKER-KEAMINLAKNPAGMNASLSVGEQLEGEKVYVISLNDNAADGRDTSWIYDADFE  359
AVFGRQETFHIGD-KECTLVLIKNPVGATQAIEMIKLAPYPFSLSVLLNANYADGIDTSWIWDADFE  365
EVAGRYRTVQVGPRHTARVLLAKNPAGWQEALSMVDRTGA--GVVIAVNGQVPDGEDLSWLWDVRFE  344
EVAGRYRTVRIGA-HQARILLAKNPAGWQEALAMVDKHAD--GVVIAVNGRVPDGEDLSWLWDVRFE  347
   ** . .:    : . : * ***.*   :: : .         : :* .  ** * **::*. **

                       III                                  IV
KLSKQQIEAIIVTGTRAEELQLRLKLAEVEVPIIVERDIYKATAKTMDYKGFT--VAIPNYTSLAPM  424
QITDMDIPEINAGGVRHSEIARRLRVTGYPAEKITETSNLEQVLKTIENQDCKHAYILATYTAMLEF  432
DFSGVQV---VAAGERGTDLAVRLGYADVEHTLVHDT------ISAIKSCPPGHVEVIANYTAFLQL  402
HFEKTRV---VAAGERGTDLAVRLGYAGVEHTLVHDT------VAAIASCPPGRVEVVANYTAFLQL  405
.:    :    . * *  ::  **  :      : :         ::          : .**::  :

LEQL------NRSFEGGQS    437
RELLASRQIVRKEMN----    447
NRRLS--------------    407
QRALARRG-----------    413
 . *
```

Supplementary Figure 1. Multiple amino acid sequence alignment of MurT from *S. aureus* (MURT_STAAN), *S. pneumoniae* (MURT_STRR6), *M. smegmatis* (A0R5Q7_MYCS2), and *M. tuberculosis* (I6Y4C7_MYCTU) using CLUSTAL version 1.2.4 (https://www.ebi.ac.uk/Tools/msa/clustalo/). The MurT central domain is displayed in black while the C-terminal domain (DUF1727) is shown in orange. The ATP and Mg^{2+} binding regions are presented in blue and green rectangles, respectively. A cysteine-rich region is shown in purple, with *S. aureus* and *S. pneumoniae* conserved cysteines in **bold**. The four conserved regions (I, II, III, and IV) of the MurT DUF1727 domain are indicated in red. The two residues and the DNAAD motif involved in the recognition and channeling of the ammonia produced by GatD, in *S. aureus*, are shown in grey.

```
CLUSTAL O(1.2.4) multiple sequence alignment

sp|A0A0H3JN63|GATD_STAAN            -------------MHELTIYHFMSDKLNLYS  18
sp|Q8DNZ8|GATD_STRR6                MVYTSLSSKDGNYPYQLNIAHLYGNLMNTYG  31
tr|A0A0D6J2S9|A0A0D6J2S9_MYCSM      ----------MTHPSTVRIGLVLPDVMGTYG  21
tr|I6XI14|I6XI14_MYCTU              ---------------MVRIGLVLPDVMGTYG  16
                                                    : *  .  : :. *.

DIGNIIALRQRAKKRNIKVNVVEINETEGITFDECDIFFIGGGSDREQALATKELSKIKTPLKEAIE  85
DNGNILMLKYVAEKLGAHVTVDIVSLHDDFDENHYDIAFFGGGQDFEQSIIADDLPAKKESIDNYIQ  98
DGGNAVVLRQRLRLRGIDAEIVEITLDDPV-PESLDLYTLGGAEDYAQRLATKHLIRH-PGLQRAAE  86
DGGNAVVLRQRLLLRGIAAEIVEITLADPV-PDSLDLYTLGGAEDYAQRLATRHLRRY-PGLQRAAG  81
* ** : *:      .  . :  :.  : .  :  *:  :**..*  * : : .*      :..

DGMPGLTICGGYQFLGKKYITPDGTELEGLGILDFYTESK-TNRLTGDIVIE--SDTFG-TIVGFEN  148
NDGVVLAICGGFQLLGQYYVEASGKRIEGLGVMGHYTLNQTNNRFIGDIKIH--NEDFDETYYGFEN  163
RGAPILAICAAIQVLGHWYETSAGERVEGVGLLDATTSPQ-EARTIGEVASRPLPDGLDQPLTGFEN  152
RGAPVLAICAAIQVLGHWYETSSGDRVDGVGLLDVTTSPQ-DARTIGELVSKPLLAGLTQPLTGFEN  147
.   *:**.. *.**: *     * .::*:*::.  *  :   *  *::  .     :     ****

HGGRTYH--DFGTLGHVTFGYGNNDEDKKEGIHYKNLLGTYLHGPILPKNYEITDYLLEKACERKG-  212
HQGRTFLSDDQKPLGQVVYGNGNNEEKVGEGVHYKNVFGSYFHGPILSRNANLAYRLVTTALKKKYG  230
HRGGTLLGSDARPLGAVTKGAGNRAGDGFDGAVQGSVVATYMHGPCLARNPQLADHLLSRVVGDLP-  218
HRGGTVLGPGTSPLGAVVKGAGNRAGDGFDGAVAGSVVATYMHGPCLARNPELADLLLSKVVGELA-  213
* * *    .   ** *. * **.  .  :*    .:..:*:*** * :* :::  *:  .

IPFEPKEIDNEAEIQAKQVLIDRANRQKKSR--     243
QDIQLPAY---EDILSQEIAEEYSDVKSKADFS     260
-PLELPEVER---LRSERL-AAPRRV-------     239
-PLDLPEVDL---LRRERL-SAR----------     231
  ::         :  :.:
```

Supplementary Figure 2. Multiple amino acid sequence alignment of GatD from *S. aureus* (MURT_STAAN), *S. pneumoniae* (MURT_STRR6), *M. smegmatis* (A0R5Q7_MYCS2), and *M. tuberculosis* (I6Y4C7_MYCTU) using CLUSTAL version 1.2.4 (https://www.ebi.ac.uk/Tools/msa/clustalo/). Two conserved regions important for glutamine amidotransferase activity are presented in red. The two conserved residues responsible for the catalytic activity (C94, H189) of GatD are indicated in blue. The Y17 residue that interacts with MurT protein is displayed in a green rectangle. The D32 of *S. pneumoniae* that substitutes the missing glutamate in the catalytic triad of GatD is shown in purple.

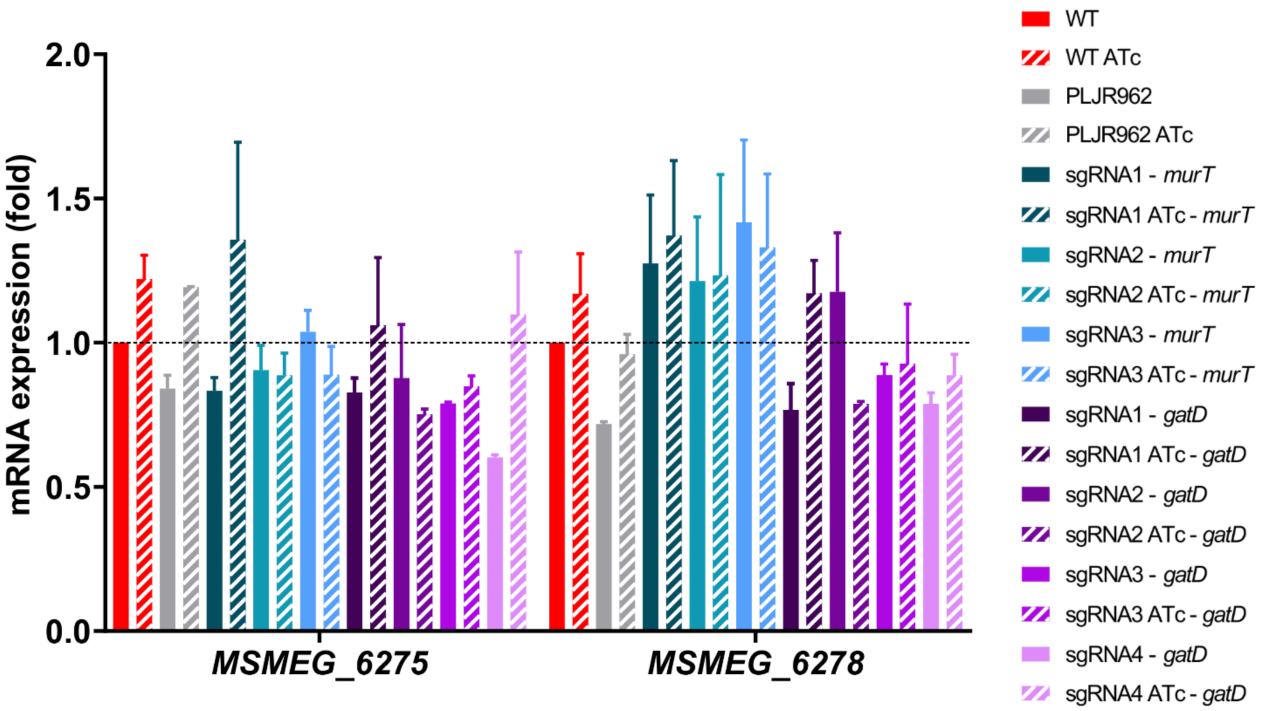

Supplementary Figure 3. Results of qRT-PCR assays for the *murT* (blue shade bars) **and *gatD*** (purple shade bars) **knockdown mutants constructed in *M. smegmatis* WT (n=2).** The graph shows the mean of the relative mRNA expression of *MSMEG_6275* and *MSMEG_6278* normalized to *sigA*, at 6 hours post-induction, with (stripped bars) and without (smooth bars) ATc. The dashed lines show the WT sample as calibrator. Error bars show the standard error of the mean. Multiple comparisons were made using one-way ANOVA. No significant differences were found.

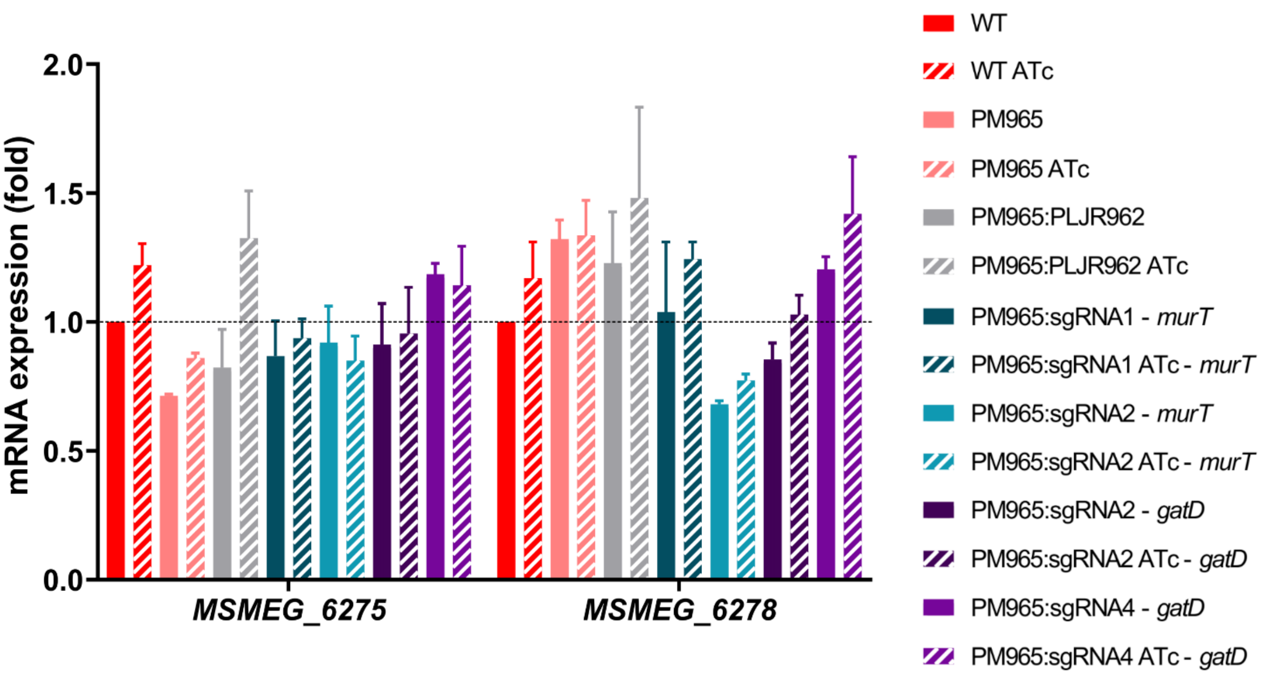

Supplementary Figure 4. Results of qRT-PCR assays for the double mutants, that is, the *murT* (blue shade bars) **and *gatD*** (purple shade bars) **knockdown mutants constructed in the *M. smegmatis* PM965 strain** (n=2). The graph shows the mean of the relative mRNA expression of *MSMEG_6275* and *MSMEG_6278* normalized to *sigA*, at 6 hours post-induction, with (stripped bars) and without (smooth bars) ATc. The dashed lines show the WT sample as calibrator. Error bars show the standard error of the mean. Multiple comparisons were made using one-way ANOVA. No significant differences were found.

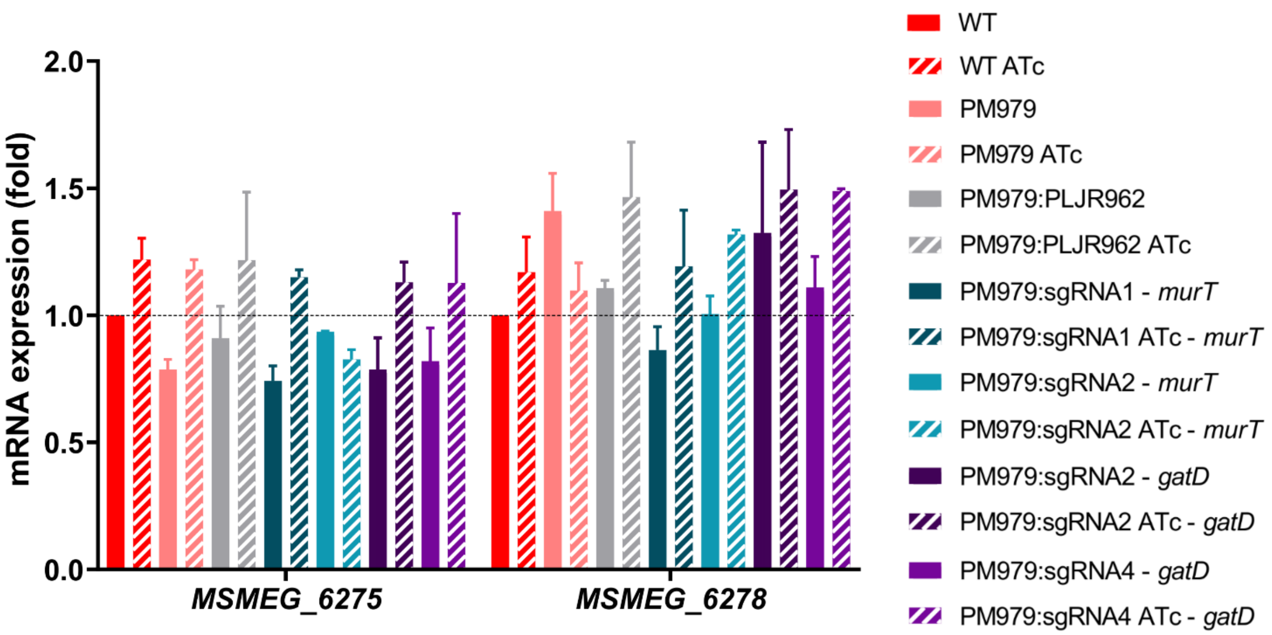

Supplementary Figure 5. Results of qRT-PCR assays for the triple mutants, that is, the *murT* (blue shade bars) **and *gatD*** (purple shade bars) **knockdown mutants constructed in the *M. smegmatis* PM979 strain** (n=2). The graph shows the mean of the relative mRNA expression of *MSMEG_6275* and *MSMEG_6278* normalized to *sigA*, at 6 hours post-induction, with (stripped bars) and without (smooth bars) ATc. The dashed lines show the WT sample as calibrator. Error bars show the standard error of the mean. Multiple comparisons were made using one-way ANOVA. No significant differences were found.

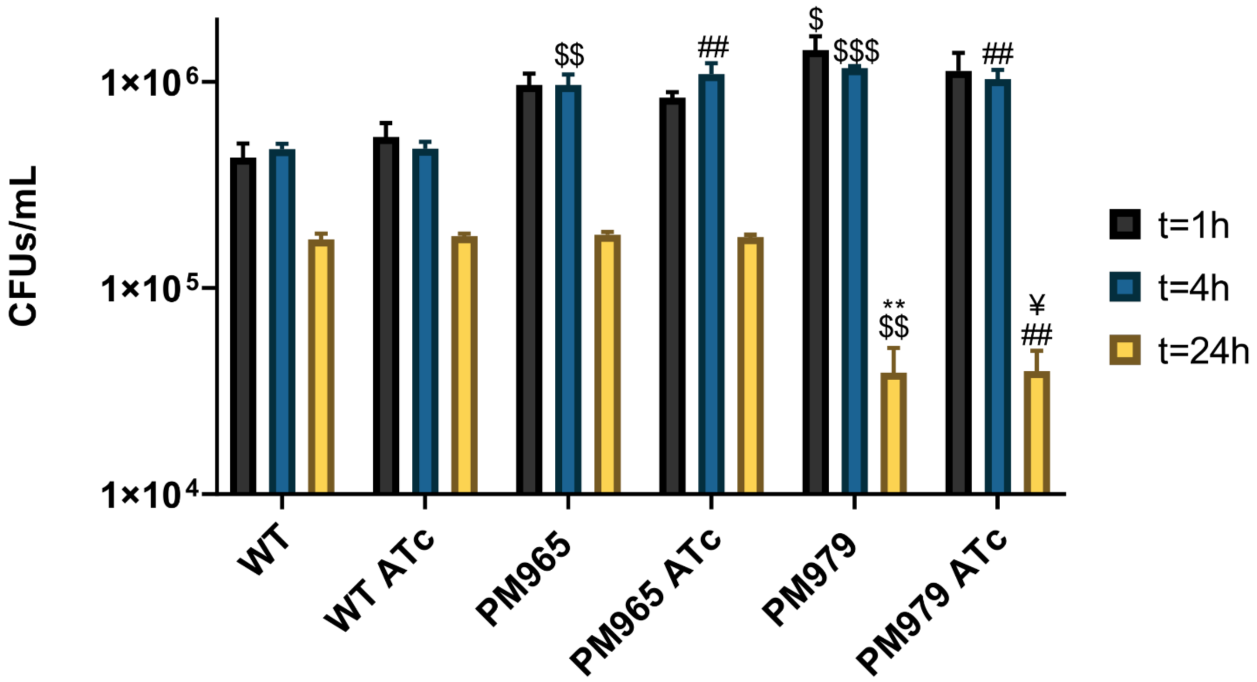

Supplementary Figure 6. Logarithmic representation of the mean of the bacterial survival of the parental strains *M. smegmatis* WT, PM965 and PM979, with and without ATc, in CFUs/mL, after infection and disruption of J774.A1 macrophages (n=3). Error bars show the standard error of the mean. Multiple comparisons were made using one-way ANOVA, with significance levels: * $P < 0.05$; ** $P < 0.01$; *** $P < 0.001$. Significant differences are indicated with symbols: $ comparing to the WT strain; # comparing to the induced WT strain (WT ATc), * comparing to the PM965 strain; ¥ comparing to the induced PM965 strain (PM965 ATc).

1.2 Supplementary Tables

Table S1 – sgRNAs used to target *murT*, *gatD* and *namH* in *M. smegmatis*. [a] - mRNA knockdown caused by the electroporation of the indicated sgRNA in the *M. smegmatis* WT strain was measured by qRT-PCR after ATc induction. Fold knockdown efficiency is relative to an uninduced control plasmid containing the same sgRNA.

Target gene	Name of the oligos	PAM	Base-pairing region of the sgRNA (the 12 bp seed region is underlined)	BLASTed sequence in the genome of *M. smegmatis* (seed region + PAM)	Fold knockdown efficiency[a]
murT	sgRNA1	5 '- GCAGAAC - 3'	5' - GCTGATCACGCGACAGATTGA - 3'	5' - GCGACAGATTGAGCAGAAC - 3'	57
	sgRNA2	5' - CAGGAAC - 3'	5' - GACAGCCGCCGGTTCAGTTG - 3'	5' - CCGGTTCAGTTGCAGGAAC - 3'	1.6
	sgRNA3	5' - GCAGGAC - 3'	5' - GCGGCCCTGGCCGAGCTGCC - 3'	5' - GGCCGAGCTGCCGCAGGAC - 3'	21.1
gatD	sgRNA1	5' - CGAGAAG - 3'	5' - GAGCGGACGTGCGTCGGAAC - 3'	5' - GTGCGTCGGAACCGAGAAG - 3'	1.7
	sgRNA2	5' - CGAGGAT - 3'	5' - ACCTGGATGGCCGCGCAGATCG - 3'	5' - CCGCGCAGATCGCGAGGAT - 3'	2.4
	sgRNA3	5' - CGAGCAG - 3'	5' - GCCTCCTGCGGAGAGGTGGTGGCGT - 3'	5' - AGGTGGTGGCGTCGAGCAG - 3'	3.3
	sgRNA4	5' - AGAGCAG - 3'	5' - GCAGATCGCCGACCACGCGGG - 3'	5' - CGACCACGCGGGAGAGCAG - 3'	2.5
namH	sgRNA1	5' - CTGGAAC - 3'	5' - AGAGTGCTTCGCCAGCCTAG - 3'	5' - TCGCCAGCCTAGCTGGAAC - 3'	7.5
	sgRNA2	5' - GAAGAAG - 3'	5' - GCTTCACCGAGTCGGTGGTCTC - 3'	5' - GTCGGTGGTCTCGAAGAAG - 3'	5.6
	sgRNA3	5' - GGGGAAG - 3'	5' - GGTTCGCGGACAACCCGTTT - 3'	5' - GACAACCCGTTTGGGGAAG - 3'	5.3

Table S2 - Primers designed and synthesized to clone the sgRNAs into PLJR962 for CRISPRi-mediated targeting in *M. smegmatis*.
[a] – PAM strength was described in accordance with the table of functional PAMs *in vivo* for $dCas9_{Sth1}$-mediated targeting in mycobacteria defined by Rock *et al.*; [b] – Calculated with the Eurofins Genomics Melting temperature (Tm) formula.

Target gene	Name of the oligos	PAM	PAM strength[a]	Gene Location 5'-3' (bp)	Primers		Length (bp)	% GC	Tm[b] (°C)
murT	sgRNA1	5 '- GCAGAAC - 3'	5	406	PFwd	5' - GGGAGCTGATCACGCGACAGATTGA - 3'	25	56	66
					PRv	5' - AAACTCAATCTGTCGCGTGATCAGC - 3'		48	63
	sgRNA2	5' - CAGGAAC - 3'	14	1220	PFwd	5' - GGGAGACAGCCGCCGGTTCAGTTG - 3'	24	67	70
					PRv	5' - AAACCAACTGAACCGGCGGCTGTC - 3'		58	66
	sgRNA3	5' - GCAGGAC - 3'	15	153	PFwd	5' - GGGAGCGGCCCTGGCCGAGCTGCC - 3'	24	83	76
					PRv	5' - AAACGGCAGCTCGGCCAGGGCCGC - 3'		75	73
gatD	sgRNA1	5' - CGAGAAG - 3'	1	498	PFwd	5' - GGGAGAGCGGACGTGCGTCGGAAC - 3'	24	71	71
					PRv	5' - AAACGTTCCGACGCACGTCCGCTC - 3'		63	68
	sgRNA2	5' - CGAGGAT - 3'	9	299	PFwd	5' - GGGAACCTGGATGGCCGCGCAGATCG - 3'	26	69	73
					PRv	5' - AAACCGATCTGCGCGGCCATCCAGGT - 3'		62	70
	sgRNA3	5' - CGAGCAG - 3'	13	383	PFwd	5' - GGGAGCCTCCTGCGGAGAGGTGGTGGCGT - 3'	29	72	77
					PRv	5' - AAACACGCCACCACCTCTCCGCAGGAGGC - 3'		66	74
	sgRNA4	5' - AGAGCAG - 3'	13	652	PFwd	5' - GGGAGCAGATCGCCGACCACGCGGG - 3'	25	76	75
					PRv	5' - AAACCCCGCGTGGTCGGCGATCTGC - 3'		68	71
namH	sgRNA1	5' - CTGGAAC - 3'	11	-44	PFwd	5' - GGGAAGAGTGCTTCGCCAGCCTAG - 3'	24	63	68
					PRv	5' - AAACCTAGGCTGGCGAAGCACTCT - 3'		54	64
	sgRNA2	5' - GAAGAAG - 3'	1	318	PFwd	5' - GGGAGCTTCACCGAGTCGGTGGTCTC - 3'	26	65	71
					PRv	5' - AAACGAGACCACCGACTCGGTGAAGC - 3'		58	68
	sgRNA3	5' - GGGGAAG - 3'	4	1142	PFwd	5' - GGGAGGTTCGCGGACAACCCGTTT - 3'	24	63	68
					PRv	5' - AAACAAACGGGTTGTCCGCGAACC - 3'		54	64

Table S3 - Primers used to quantify the mRNA expression levels of target genes by qRT-PCR in *M. smegmatis*. [a] - qRT-PCR primers were designed to avoid primer secondary structures and sequence repeats, have at least 50% of GC content and have a Tm between 57-63 °C; [b] - Calculated with the Eurofins Genomics Tm formula; [c] – AE designates the amplification efficiency calculated for each pair of primers in a single-run qPCR amplification of the calibrator sample (*M. smegmatis* WT), performed in triplicates.

**qRT-PCR primers[a]**							
**Target gene**		**Primer sequences**	**Length (bp)**	**% GC**	**Tm[b] (° C)**	**Product size (bp)**	**AE[c] (%)**
***sigA* (*MSMEG_2758*)**	PFwd	5' - ACACCGACCTGGAACTCG - 3'	18	61	58	152	80
	PRv	5' - GACGCCTTGTCCTTCTCG - 3'	18	61	58		
***murT* (*MSMEG_6276*)**	PFwd	5' - AATCGACCACCACCAGGAT - 3'	19	53	57	213	105
	PRv	5' - CGACAGATTGAGCAGAACGA - 3'	20	50	57		
***gatD* (*MSMEG_6277*)**	PFwd	5' - GCGACGGTTTCGACGGTG - 3'	18	67	61	167	95
	PRv	5' - AGGCGCTCAGAACGCAAAC - 3'	19	58	59		
MSMEG_6275	PFwd	5' - GAAGCTGTAGTCGAACCCCG - 3'	20	60	61	160	84
	PRv	5' - CTCAACCCTGGTGTCGATCC - 3'	20	60	61		
MSMEG_6278	PFwd	5' - TTGAACACCCCGACGATCAC - 3'	20	55	59	154	80
	PRv	5' - CGATGTCCTCGACTTCGCA - 3'	19	58	59		
***namH* (*MSMEG_6410*)**	PFwd	5' - CGTTCTTCAAGTGCCTCACC - 3'	20	55	59	165	87
	PRv	5' - GTTGCCTTCCACCACACC - 3'	18	61	58		
MSMEG_6409	PFwd	5' - CCGCAGCTTCGAGATCAAGG - 3'	20	60	61	137	83
	PRv	5' - CCGGTACAGGTTCTTCGGAC - 3'	20	60	61		
MSMEG_6411	PFwd	5' - GTGCCAGAAGCTGTGACCG - 3'	19	63	61	120	85
	PRv	5' - GGGATCACCTTCGATGTGCC - 3'	20	60	61		

Table S4 – The EUCAST non-species related PK/PD breakpoints for amoxicillin (AMX), amoxicillin-clavulanate (AMX+CLA), cefotaxime (CTX) and meropenem (MEM). These breakpoints, used when there are no species-specific breakpoints, are based on version 12 of the EUCAST guidelines on susceptibility testing (https://www.eucast.org/clinical_breakpoints/). For the AMX+CLA breakpoint, the concentration of clavulanate used was fixed at 2 µg/mL.

EUCAST breakpoints (µg/mL)		
	S ≤	R >
**AMX**	2	8
**AMX+CLA**	2	8
**CTX**	1	2
**MEM**	2	8

Table S5 - Single nucleotide polymorphisms (SNPs) found in the target genes *murT*, *gatD* or *namH* through the whole genome sequencing of 172 clinical strains of *Mtb*, with the description ID of the strains in which mutations were found. Locus tags, gene names, products and positions refer to the H37Rv reference genome (AL123456.3).

Locus Tag	Gene Name	Genomic Position	Mutation	Type of Variant	Effect in Product	Frequency	Strains
Rv3712	*murT*	4158032	A>C	Missense	Lys351Thr	1/172	PT_TB0303
Rv3713	*gatD*	4158361	A>G	Synonymous	Pro45Pro	1/172	PT_TB0303
		4158493	C>T	Synonymous	Ile89Ile	10/172	PT_TB0067; PT_TB0057; PT_TB0029; PT_TB0069; PT_TB0328; PT_TB0319; PT_TB0276; PT_TB0265; PT_TB0288; PT_TB0214
		4158865	G>A	Synonymous	Ala213Ala	4/172	PT_TB0311; PT_TB0314; PT_TB0305; PT_TB0242
Rv3818	*namH*	4282589	T>C	Synonymous	Ala47Ala	1/172	PT_TB0245
		4282707	C>G	Missense	Pro87Ala	1/172	PT_TB0070
		4283319	G>A	Missense	Ala291Thr	2/172	PT_TB0313; PT_TB0250
		4283424	G>C	Missense	Ala326Pro	1/172	PT_TB0350

Table S6 - Information of the strain ID, Sequence Read Archive (SRA)/European Nucleotide Archive (ENA) accession numbers, drug-resistance profile and *in silico* TB-profiler predictions of lineages, spoligotypes and regions of difference (RD) of the clinical isolates of *Mtb* for which mutations in the target genes *murT*, *gatD* or *namH* were found through whole genome sequencing.

Strain ID	SRA/ENA Accession #	Classification	Drug-resistance Profile	TB profiler prediction		
				Lineage	Spoligotype	Regions of difference (RD)
PT_TB0303	ERR7764425	Susceptible	Susceptible	lineage6	AFRI 1	RD702
PT_TB0067	ERR2864255	Resistant	MDR	lineage2.2.1	Beijing-RD181	RD105; RD207; RD181
PT_TB0057	ERR2864286	Resistant	MDR	lineage2.2.1	Beijing-RD181	RD105; RD207; RD181
PT_TB0029	ERR2864231	Resistant	MDR	lineage2.2.1	Beijing-RD181	RD105; RD207; RD181
PT_TB0069	ERR2864228	Resistant	MDR	lineage2.2.1	Beijing-RD181	RD105; RD207; RD181
PT_TB0328	ERR7764343	Susceptible	Susceptible	lineage2.2.1	Beijing-RD181	RD105; RD207; RD181
PT_TB0319	ERR7764417	Susceptible	Susceptible	lineage2.2.1.1	Beijing-RD150	RD105; RD207; RD181; RD150
PT_TB0276	ERR7764390	Susceptible	Susceptible	lineage2.2.1	Beijing-RD181	RD105; RD207; RD181
PT_TB0265	ERR7764438	Susceptible	Susceptible	lineage2.2.1	Beijing-RD181	RD105; RD207; RD181
PT_TB0288	ERR7764362	Susceptible	Susceptible	lineage2.2.1	Beijing-RD181	RD105; RD207; RD181
PT_TB0214	ERR4781456	Resistant	Pre-XDR	lineage2.2.1.1	Beijing-RD150	RD105; RD207; RD181; RD150
PT_TB0311	ERR7764372	Susceptible	Susceptible	lineage4.8	T1; T2; T3; T5	RD219
PT_TB0314	ERR7764364	Susceptible	Susceptible	lineage4.8	T1; T2; T3; T5	RD219
PT_TB0305	ERR7764375	Resistant	Mono-DR	lineage4.8	T1; T2; T3; T5	RD219
PT_TB0242	ERR7764378	Susceptible	Susceptible	lineage4.8	T1; T2; T3; T5	RD219
PT_TB0245	ERR7764426	Susceptible	Susceptible	lineage4.1.2.1	T1; H1	RD182
PT_TB0070	ERR2864232	Resistant	MDR	lineage3	CAS	RD750
PT_TB0313	ERR7764325	Susceptible	Susceptible	lineage4.8	T1; T2; T3; T5	RD219
PT_TB0250	ERR7764333	Susceptible	Susceptible	lineage4.8	T1; T2; T3; T5	RD219
PT_TB0350	ERR7764370	Resistant	MDR	lineage4.3.4.2	LAM1; LAM4; LAM11	RD174
